# Efficient tumor synergistic chemoimmunotherapy by self-augmented ROS-responsive immunomodulatory polymeric nanodrug

**DOI:** 10.1186/s12951-023-01842-1

**Published:** 2023-03-16

**Authors:** Jinxiao Song, Mingyang Cheng, Yi Xie, Kangkang Li, Xinlong Zang

**Affiliations:** School of Basic Medicine, Qingdao University, Ningxia Road 308, Qingdao, People’s Republic of China

**Keywords:** Stimuli-responsive, Prodrug copolymer, Immunogenic cell death, Immunosuppression tumor microenvironment, Indoleamine 2,3-dioxygenase

## Abstract

Immunotherapy has emerged as a promising therapeutic strategy for cancer therapy. However, the therapeutic efficacy has been distracted due to poor immunogenicity and immunosuppressive tumor microenvironment. In this study, a self-augmented reactive oxygen species (ROS) responsive nanocarrier with immunogenic inducer paclitaxel (PTX) and indoleamine 2,3-dixoygenase 1 (IDO1) blocker 1-methyl-d, L-tryptophan (1-MT) co-entrapment was developed for tumor rejection. The carrier was composed of poly (ethylene glycol) (PEG) as hydrophilic segments, enzyme cleavable 1-MT ester and ROS-sensitive peroxalate conjugation as hydrophobic blocks. The copolymer could self-assemble into prodrug-based nanoparticles with PTX, realizing a positive feedback loop of ROS-accelerated PTX release and PTX induced ROS generation. Our nanoparticles presented efficient immunogenic cell death (ICD) which provoked antitumor immune responses with high effector T cells infiltration. Meanwhile immunosuppressive tumor microenvironment was simultaneously modulated with reduced regulatory T cells (Tregs) and M2-tumor associated macrophages (M2-TAMs) infiltration mediated by IDO inhibition. The combination of PTX and 1-MT achieved significant primary tumor regression and reduction of lung metastasis in 4T1 tumor bearing mice. Therefore, the above results demonstrated co-delivery of immunogenic inducer and IDO inhibitor using the ROS amplifying nanoplatform with potent potential for tumor chemoimmunotherapy.

**Supplementary Information:**

The online version contains supplementary material available at 10.1186/s12951-023-01842-1.

## Introduction

Immunotherapy has emerged as a powerful therapeutic strategy for cancer treatment, which could kill tumor cells through host immune system with long protection effects. However, the lack of immunogenicity in tumor leads to insufficient immune responses [1–3]. Immunosuppressive tumor microenvironment further promotes tumor cells escape from antitumor immunity and conventional therapeutical strategies such like chemotherapy [4].

Immunogenic cell death (ICD) is a particular modality of cell death that can be induced by some specific chemotherapeutics such as doxorubicin (DOX), mitoxantrone (MIT) and paclitaxel (PTX) [5–9]. Cancer cells undergoing immunogenic cell death are characterized with the release of danger associated molecular patterns (DAMPs), such as calreticulin (CRT), adenosine triphosphate (ATP) and high mobility group box 1 (HMGB1), etc. [10–12]. These patterns can reinforce antitumor immune responses through dendritic cells (DCs) activation and intratumoral cytotoxic T lymphocyte recruitment [13]. However, ICD alone cannot generally make for good therapeutic outcome as expected. One reason is these immunogenic inducers’ poor assess to tumor location, revealing weak antitumor immunity [14]. Increasing doses alone can’t fortunately solve the problem, which may result in off-target cytotoxicity to heart, liver and kidney associated with high dosage and unspecific distribution [15, 16]. Another is that these agents, severing as a “double-edged sword”, can trigger detrimental immunosuppression and cripple ICD mediated immune responses [15, 17]. Herein, it is essential to promote immunogenicity meanwhile reverse immunosuppressive tumor microenvironment for more efficacious antitumor therapy. For instance, doxorubicin or PTX combination with PD-L1 (siRNA or antibody) showed excellent performances in synergistic chemoimmunotherapy through promoting tumor immunogenicity and blockading PD-1/PD-L1 axis [18, 19]. Photodynamic therapy (chlorine e6), in combination with anti-lactic acid (lonidamine) and anti-β-TGF receptor (SB505124) suppressed distant and metastatic tumor growth through ternary regulation [20]. These studies underline the fact that the synergetic modulation of tumor immunogenicity and immunosuppression can facilitate therapeutic efficacy in cancer treatment.

The central event underlying immunosuppressive tumor microenvironment is upregulated indoleamine-2.3-dioxygenase 1 (IDO1) that can metabolize tryptophan (Trp) into kynurenine (Kyn). IDO1 overexpression promoted the differentiation of DCs toward an immunosuppressive phenotype, resulting in a robust expansion of regulatory T cells (Tregs) [21, 22]. IDO1 can also orchestrate local immune suppression by Tregs dependent intratumoral infiltration of tumor associated macrophages (TAMs) and myeloid-derived suppressor cells (MDSCs) [23, 24]. These tryptophan metabolites inhibited effector T cells proliferation and promoted their apoptosis. As a result, the high expression of IDO1 was consistently associated with poor prognosis in patients with solid tumors [25]. Hence, blocking IDO pathway has been considered as an effective strategy to revive immunosurveillance. IDO1 inhibitors such as 1-methyl-D, L-tryptophan (1-MT, NLG8189) and navoximod (NLG919), can effectively inhibit IDO enzymes and relieve tumor immunosuppression [25–28]. Recently, doxorubicin and 1-MT achieved synergistic tumor chemotherapy, which abolished primary tumor growth and eradicated metastatic lesions [26]. Huang et al. reported a laser/GSH activatable polymeric carrier with oxaliplatin and phthalocyanine mediated ICD effect and IDO blockage of NLG919, which demonstrated enhanced cancer immunotherapy [29, 30]. Therefore, co-delivery of ICD inducer and IDO inhibitor might suppress tumor growth by synchronously boosting immunity and intervening immunosuppression in tumor microenvironment.

**Scheme 1.**
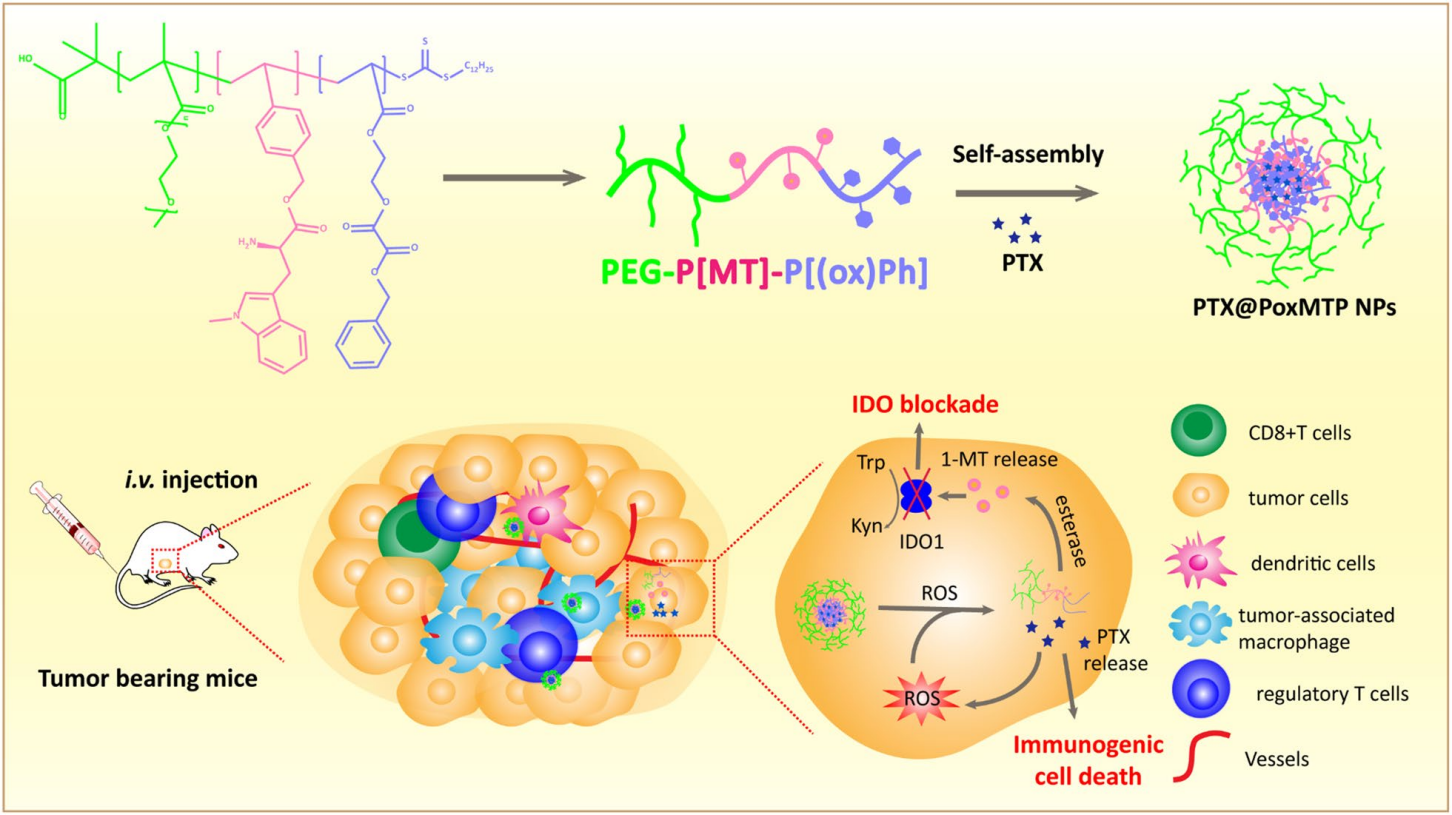
Schematic illustration of the formation of ROS-augmented nanocarrier driven by self-assembly in aqueous solution. PTX-mediated ROS generation further “fuels” nanoparticle disassembly and PTX release to promote tumor immunogenicity. Meanwhile esterase responsive 1-MT release intervenes IDO mediated immunosuppressive tumor microenvironment

In this study, we developed a ROS-responsive nanodrug with chemical conjugate with IDO1 inhibitor 1-MT and physical encapsulation of ICD inducer PTX for tumor synergetic chemoimmunotherapy. The polymeric nanocarrier is composed of polyacrylate derivatives (P[Ph(ox)]-P[MT]-PEG), which consists of hydrophilic poly (ethylene glycol), enzyme cleavable 1-MT ester and oxidation-sensitive phenyl peroxalate ester. As illustrated in Scheme 1, the immunostimulatory prodrug-based nanoparticles (PTX@PoxMTP NPs) are stable in circulation and then highly accumulate in tumor through passive targeting after intravenous administration. After endocytosis into tumor cells, intracellular reactive oxygen species (ROS) triggers PTX release to induce ICD and subsequentially boost immune responses. In this process, PTX-mediated ROS generation further “fuel” nanoparticle disassembly and PTX release, which forms a positive feedback loop to promote tumor immunogenicity. 1-MT was taken off from the polymeric carrier in the presence of esterase and efficiently intervened IDO1 mediated immunosuppressive tumor microenvironment. Notably, co-delivery of PTX and 1-MT demonstrated significant improvements in antitumor immunity with enriching CD8 + T cells and dampening immunosuppressive cell components, thereby efficiently reducing primary tumor burden and metastatic lesions. In summary, the self-augmented ROS-responsive nanocarrier was with potent potential for tumor chemoimmunotherapy by immunogenic amplification and immunosuppressive modulation.

**Scheme 2.**
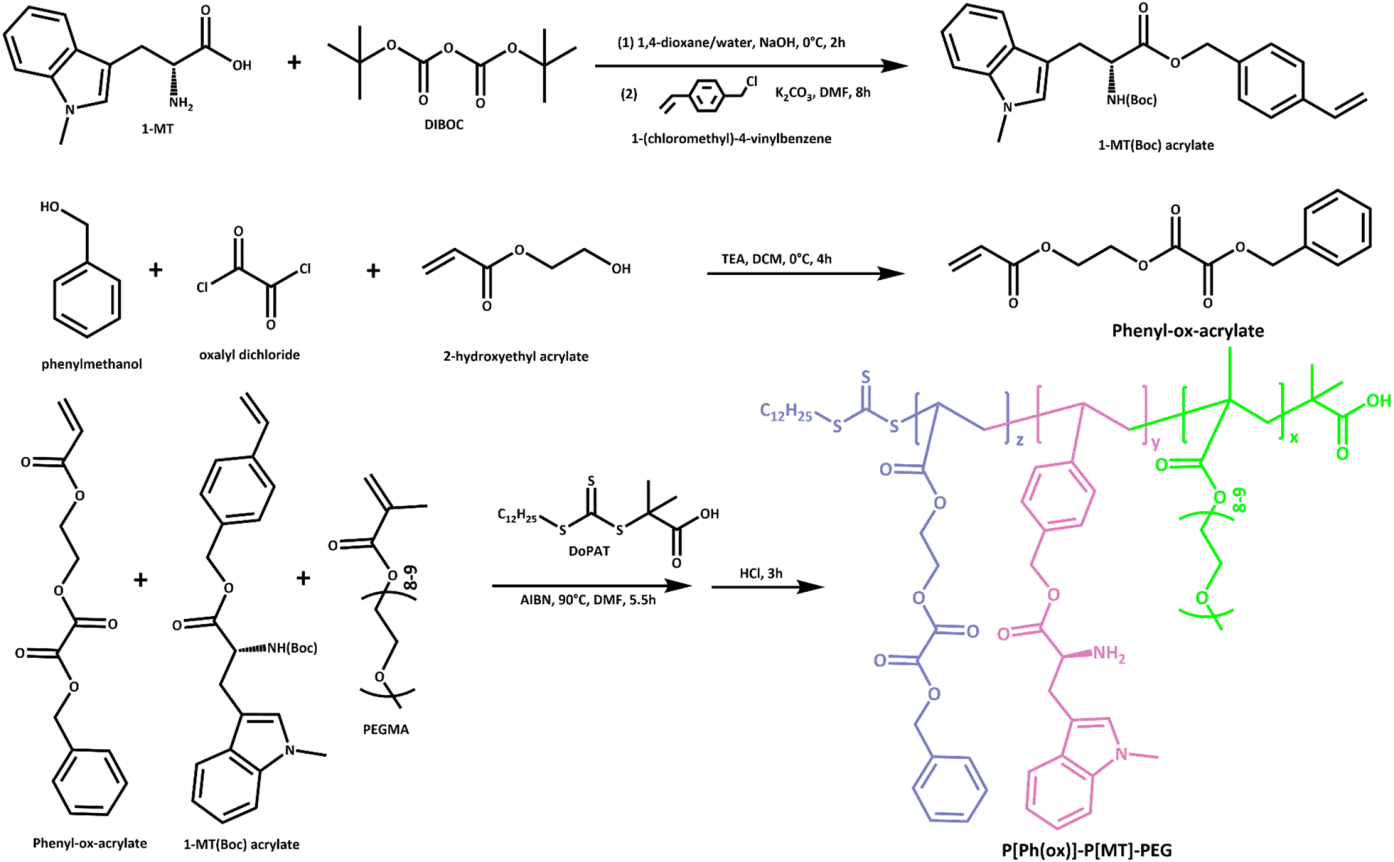
Synthesis of the amphilic prodrug-based copolymer

## Materials and methods

### Materials

All chemical regents were purchased from commercial sources. 2-Hydroxyethyl methacrylate (2-HEMA), 2-methoxyethyl methacrylate (PEGMA), 2-(Dodecylthiocarbonothioylthio) propionic acid (DoPAT), 1-(chloromethyl)-4-vinylbenzene, phosphotungstic acid (PTA), oxalyl chloride, phenylmethanol, 1-MT and PTX were obtained from Energy Chemical Co. Ltd (Shanghai, China). Nile red and 1,1′-Dioctadecyl-3,3,3′,3′-tetraMethylindotricarbocyanine iodide (DiR) was obtained from Dalian Meilun Biotechnology Co. Ltd (China). 4′,6-diamidino-2-phenylindole (DAPI), BCA protein determination kit, RIPA lysate and MTT solution were from shanghai Solaibio Co. Ltd (China). Annexin V-FITC/PI and PI staining, ROS (DCFH-DA), HMGB1, CRT and ATP assay Kits were obtained from Shanghai Beyotime Institute of Biotechnology Co. Ltd (China). Anti-mouse 1DO1 and β-actin antibodies were obtained from Rosemont Proteintech Co. Ltd (Wuhan, China). Immune cells staining kits were purchased from Hangzhou Multiscience Biotechnolgoy (China). All other chemical regents were obtained from Sinopharm Chemical Reagent (Shanghai, China) without further purification.

### Cell lines and animals

Human MDA-MB-231 and murine 4T1 cells were obtained from American Type Culture Collection (ATCC, USA). Tumor cells were cultured in RMPI1640 medium containing 10% fetal bovine serum and 1% antibiotics in a humanified incubator at 37 °C with 5% CO_2_.

Balb/c female mice were purchased from Jinan Pengyue Co. Ltd (Jinan, China) and supplied with enough food and water. All experimental procedures were conducted with the guidelines built by the Institutional Animal Care and Use Committee of Qingdao University. The committee has approved all our animal experiments.

### Synthesis of P[Ph(ox)]-P[MT]-PEG

#### Synthesis of 1-MT(Boc) acrylate

The amine of 1-MT was protected with DIBOC through a simple amidation reaction as our previous report [31]. As illustrated in Scheme 2, 0.5 g 1-MT and 0.75 g DIBOC were dissolved into 1,4-dioxane-water (1:1, v/v). The mixture was cold to 0 °C and 0.2 g NaOH was added and stirred for 2 h. 3 M HCl was added into the above system till pH4-5 and then mixed with EtOAc. After removing the solvent, 1-MT-Boc was dissolved into 10 mL DMF with 1-(chloromethyl)-4-vinylbenzene and mixed with anhydrous K_2_CO_3_ (0.8 g). The reaction was performed at 80 °C for 8 h and then passed through gel column with EtOAc/PET = 1:40 (v/v). The solvent was removed and 1-MT(Boc) acrylate was obtained.

#### Synthesis of Phenyl-ox-acrylate

Phenylmethanol (1.08 g) and 2-HEMA (1.3 g) were placed into a round bottom flask with 10 mL anhydrous DCM and catalytic TEA. Subsequently, oxalyl chloride (1.2 mL) was dropwise added and the system was stirred at 0 °C under N_2_ atmosphere. After removing the solvent, the crude product (phenyl-ox-acrylate) was redissolved into DMF without further purification.

#### Synthesis of P[Ph(ox)]-P[MT]-PEG

Phenyl-ox-acrylate (147.6 mg), 1-MT(Boc) acrylate (157 mg) and PEGMA (500 mg) were added into a Schlenk tube with 10 mL DMF. Meanwhile RAFT initiator DoPAT and catalytic AIBN were mixed with above system. Triple freeze-pump-thawing cycles were performed to remove oxygen and moisture and then the mixture was stirred at 90 °C for 5 h under the protection of nitrogen. After quenching the reaction, HCl/EtOAc was dropwise added and stirred at room temperature for 3 h. Finally, the system was precipitated into cold ethyl ether three times and dried under vacuum to obtain brown powder of P[Ph(ox)]-P(MT)-PEG.

#### Characterization of P[Ph(ox)]-P[MT]-PEG

The chemical structure of P[Ph(ox)]-P[MT]-PEG and intermediate products were characterized using ^1^H NMR spectrum (Bruker AVANCE III HD 400 MHz, Switzerland). The hemolytic activity was also investigated through incubation with fresh rabbit red blood cells. Briefly, fresh rabbit blood was diluted to a final concentration of 10% red blood cells which were incubated with equivalent polymer solutions with concentration ranging from 0.1 to 2 mg/mL. While distilled water was selected as positive control. The samples were treated in shake incubator at 37 °C for 2 h. The absorbance of released hemoglobin was determined at the wavelength of 540 nm in a microplate reader (BioTek, USA) and the hemolysis was calculated by the following formula:

$${\text{Hemolytic ratio}}(\% ) = \frac{{A_{{{\text{sample}}}} \_A_{{{\text{nagative}}}} }}{{A_{{{\text{water}}}} \_A_{{{\text{negative}}}} }} \times 100\%$$

where *A*_sample_*, A*_negaive_ and *A*_water_ represent the absorbance of sample, saline and positive sample (water) at 540 nm, respectively.

### Preparation and characterization of PTX@PoxMTP NPs

The thin-film hydration method was adopted to prepare PTX@PoxMTP NPs as widely described [32]. In brief, P[Ph(ox)]-P(MT)-PEG (10 mg) and PTX (1 mg) were dissolved in 10 mL DCM and the solvent was removed at 35 °C to obtained a transparent film. 5 mL PBS (0.01 M, pH7.4) was added at 60 °C and the suspension was treated with ultrasound bath for 10 min followed by filtration through a 0.22 µm filter to obtain PTX@PoxMTP NPs. Likewise, blank nanoparticles (PoxMTP NPs) were prepared using the same method in the absence of PTX. PTX was also loaded into poly(ethylene glycol)-poly(ε-caprolactone) nanoparticles (PTX@PEG-PCL) by the above method for in vivo study.

Physicochemical properties have been proven to have significant influence on in vivo biodistribution and therapeutic efficacy of nanomedicines. As a result, particle size, zeta potential, morphology, drug encapsulation and release behavior were characterized. The diameter and surface charge of these nanoparticles were measured using dynamic light scattering (DLS) technique on Malvern Nano-ZS device (UK). Transmission electron microscopy (TEM) was utilized to observe the morphology. Briefly, the nanosuspensions were dropped onto copper mesh and stained with 2% PTA solution followed by photographed via TEM (JEM-2100, JEOL, JP). To evaluated the ROS-responsive properties, PTX@PoxMTP NPs were incubated with 1 mM H_2_O_2_ and then characterized using DLS technique and TEM.

To investigate the encapsulation (EE) and drug loading efficiency (DLE) of PTX into the nanoparticles, HPLC method was adopted as previously described [33]. Briefly, PTX@PoxMTP NPs were dissolved or/and swelled in methanol and the concentration of PTX was measured on Waters HPLC system (Hclass plus, Singapore). The EE and DL of PTX was calculated by the following equations:

$${\text{Encapsulation efficiency }}(\% ) = \frac{{W_{{\text{loaded PTX}}} }}{{W_{{\text{feeded PTX}}} }} \times 100\%$$

$${\text{Drug loading efficiency (\% ) = }}\frac{{W_{{\text{loaded PTX}}} }}{{W_{{{\text{nanoparticle}}s}} }} \times 100\%$$

where* W*_loaded PTX_, *W*_feeded PTX_ and *W*_nanoparticles_ represent the weight of loaded and feeded PTX, nanoparticles, respectively.

In vitro release behavior of PTX@PoxMTP NPs was investigated using dialysis method as described everywhere [34]. Briefly, PTX, 1-MT, PoxMTP NPs and PTX@PoxMTP NPs were respectively introduced into a dialysis bag (MW, 3500 Da), which was immersed into 50 mL fresh PBS containing 0.5% Tween80 in presence of 1 mM H_2_O_2_ and ester enzymes or not. At designed intervals, the samples were obtained and replenished with fresh release medium. The concentration of PTX and 1-MT concentration were determined using HPLC method as described above.

### In vitro cellular uptake

The cellular uptake of PTX@PoxMTP NPs was analyzed by flow cytometry and confocal laser scanning microscopy (CLSM) and nile red (NR) served as a fluorescence probe to label these nanoparticles. For flow cytometry, MDA-MB-231 and 4T1 cells were seeded into 6-well plates at a density of 5 × 10^5^ cells/per well and cultured overnight. The culture medium was replaced with fresh medium containing NR labelled nanoparticles and further incubated for different times. After removing the medium, tumor cells were trypsined, harvested and analyzed by flow cytometry. For optical analysis, 3 × 10^5^/well MBA-MB-231 and 4T1 cells were seeded into 6-well plate and cultured for 24 h. After discarding the culture medium, tumor cells were replenished with NR labelled nanoparticles and further incubated for different time. Subsequentially, tumor cells were washed with cold PBS three times, fixed with 4% paraformaldehyde and stained with DAPI followed by CLSM observation.

### In vitro cytotoxicity

MTT assay was firstly conducted to evaluate the cytotoxicity of different PTX formulations to tumor cells. MDA-MB-231 and 4T1 cells were seeded into 96-well plates at a density of 1 × 10^4^ cells per well and cultured overnight, respectively. PTX, PoxMTP NPs and PTX@PoxMTP NPs were added and further incubated for another 24 h. Next, 10 µL MTT solution (10 mg/mL) was dropped into each well. After 4 h incubation, 150 µL DMSO was used to dissolve the formazan crystals and the absorbance was measured at 570 nm in a microplate reader (SynergyMx, BioTek, USA). The cell viability was calculated by the following equation meanwhile half inhibition concentration (IC_50_) was calculated using Graphpad prism software (San Diego, CA, USA).

$${\text{Cell survival}}(\% ) = \frac{{A_{{{\text{treated}}}} \_A_{{{\text{blank}}}} }}{{A_{{{\text{control}}}} \_A_{{{\text{blank}}}} }} \times 100\%$$

where *A*_treated_, *A*_blank_ and *A*_control_ were the absorbance of treatment, blank and control group at 570 nm, respectively.

### Cell cycle and apoptosis assay

Cell cycle was investigated through PI staining and analyzed by flow cytometry. 5 × 10^5^ MDA-MB-231 and 4T1 cells were seeded into 6-well plates and cultured overnight prior to different formulation treatments. Subsequently, tumor cells were harvested and DNA contents were measured via standard PI staining and flow cytometry analysis. For apoptosis assay, tumor cells pretreated with different formulations were trypsined, harvested and marked using Annexin V-PI staining and finally analyzed by flow cytometry.

### In vitro ROS detection

MDA-MB-231 and 4T1 cells were seeded into 6-well plate with a density of 5 × 10^5^ cells per well. These cells were then treated with different formulations for 48 h. Subsequently, the medium was replaced with fresh medium containing 10 µg/mL DCFH-DA and incubated for 0.5 h. After removing the medium, the cells were fixed with 4% paraformaldehyde and analyzed by flow cytometry.

### IDO1 inhibition

To evaluate the inhibition effects of different formulations, MDA-MB-231 and 4T1 cells were incubated with 50 ng/mL IFN-γ to stimulate IDO expression prior to different preparation treatments for 24 h. The supernatants were collected and the concentration of Kyn was determined by HPLC (water: acetonitrile = 90:10 with 0.1% TFA and acetic acid) at 355 nm. Meanwhile tumor cell harvest, protein extraction and immunoblot assay were performed to evaluate IDO1 expression according to standard optional protocols.

### ICD induction

MDA-MB-231 and 4T1 cells were seeded into 6-well plates and incubated with different formulations. For CRT and HMGB1 detection, MDA-MB-231 and 4T1 cells were treated with 4% paraformaldehyde and stained with CRT or HMGB1 antibodies at 4 °C overnight. After incubation with fluorescence labelled secondary antibodies, tumor cells were then analyzed by flow cytometry. Meanwhile, the culture medium was collected for ATP analysis using ELISA Kits in accordance with manufactures’ instructions.

### Dendritic cell maturation

Bone marrow-derived dendritic cells (BMDCs) were obtained from the femurs and tibiae of female Balb/c cells as described by Chen et al. [35]. BMDCs were cultured in complete RMPI1640 medium (10% FBS) containing 10 ng/mL GM-CSF. 4T1 cells pretreated with different formulations were co-cultured with above immature BDMCs for 24 h. Thereafter, BMDCs were incubated with antibodies against CD80, CD86 and MHC II, analyzed by flow cytometry.

### In vivo biodistribution

DiR labelled nanoparticles were formulated and injected into 4T1 tumor bearing mice for biodistribution evaluation. To establish 4T1 orthotopic tumor model, 1 × 10^7^ tumor cells were subcutaneously seeded into the right mammary pads. Once tumor volume upon 100 mm^3^, DiR labelled nanoparticles were intravenously administrated through tail vein injection. At designed intervals, fluorescence signals were monitored using in vivo imaging system (Carestream Molecular Imaging, USA). After 48 h administration, main organs and tumor tumors were excised, harvested and observed using the system after mice sacrifice.

### In vivo antitumor effect

For antitumor evaluation in vivo, 4T1 tumor bearing mice model was established as described above. On 7th tumor inoculation, the mice (n = 10) were administrated through tail vein injection with designed groups: saline, PTX@PEG-PCL, PoxMTP NPs and PTX@PoxMTP NPs. The injections were performed every two days and mice received five treatments. In this period, tumor size and body weight were monitored at predetermined time. At the 19th day, mice in different treatment groups were sacrificed and tumors were dissected, weighted and fixed into 4% paraformaldehyde for further experiments. Tumor volume and inhibition ratio (TGI) was calculated using the following equations:

$${\text{Tumor volume (mm}}^{3} {) = }\frac{{{\text{a}} \times {\text{b}}^{2} }}{2}$$

where “a” and “b” were the longest and shortest diameters in mice received different treatments.

$${\text{Tumor inhibition ratio (\% ) = }}\frac{{W_{{{\text{Salin}}}} \_W_{{{\text{Treated}}}} }}{{W_{{{\text{Salin}}}} }} \times 100{\text{\% }}$$

where *W*_Saline_ and *W*_Treated_ were the weight of tumors obtained from control and other treatment groups.

### HE and ICH staining

To examine the histopathological changes, tumors obtained above were embedded in paraffin and cut into 4 µm sections. These slides were treated with primary antibodies against CRT, IDO1 and Ki67 followed by secondary antibody incubation and DAPI staining. Additionally, HE staining was carried out in tumor and main organ sections. These results were visualized using Nikon Eclipsw C1 microscopy (JP).

### In vivo immune responses

For the detection of immune cell components, tumor tissues in different groups were cut into 2 mm × 2 mm pieces and incubated with digestive solution (3% collagenase IV and 0.5% DNase I) at 37 °C for 2 h. The cell suspensions were stained with antibody cocktails: DCs (FITC-CD11c, PE-CD80 and APC-CD86), tumor-associated macrophages (FITC-F4/80 and PE-CD206), CD8 + T cells (FITC- CD8) and Tregs (CD4 + CD25 + FoxP3 +) and analyzed using flow cytometry.

### Statistical Analysis

All experiments were performed at lease in triple and the results were indicated as mean ± SD. One-way ANOVA and Two-tailed Student’s *t*-test were used for statistical comparison. “P < 0.05” was considered to have statistical significance.

## Results and discussion

### Synthesis and characterization of copolymer

**Fig. 1:**
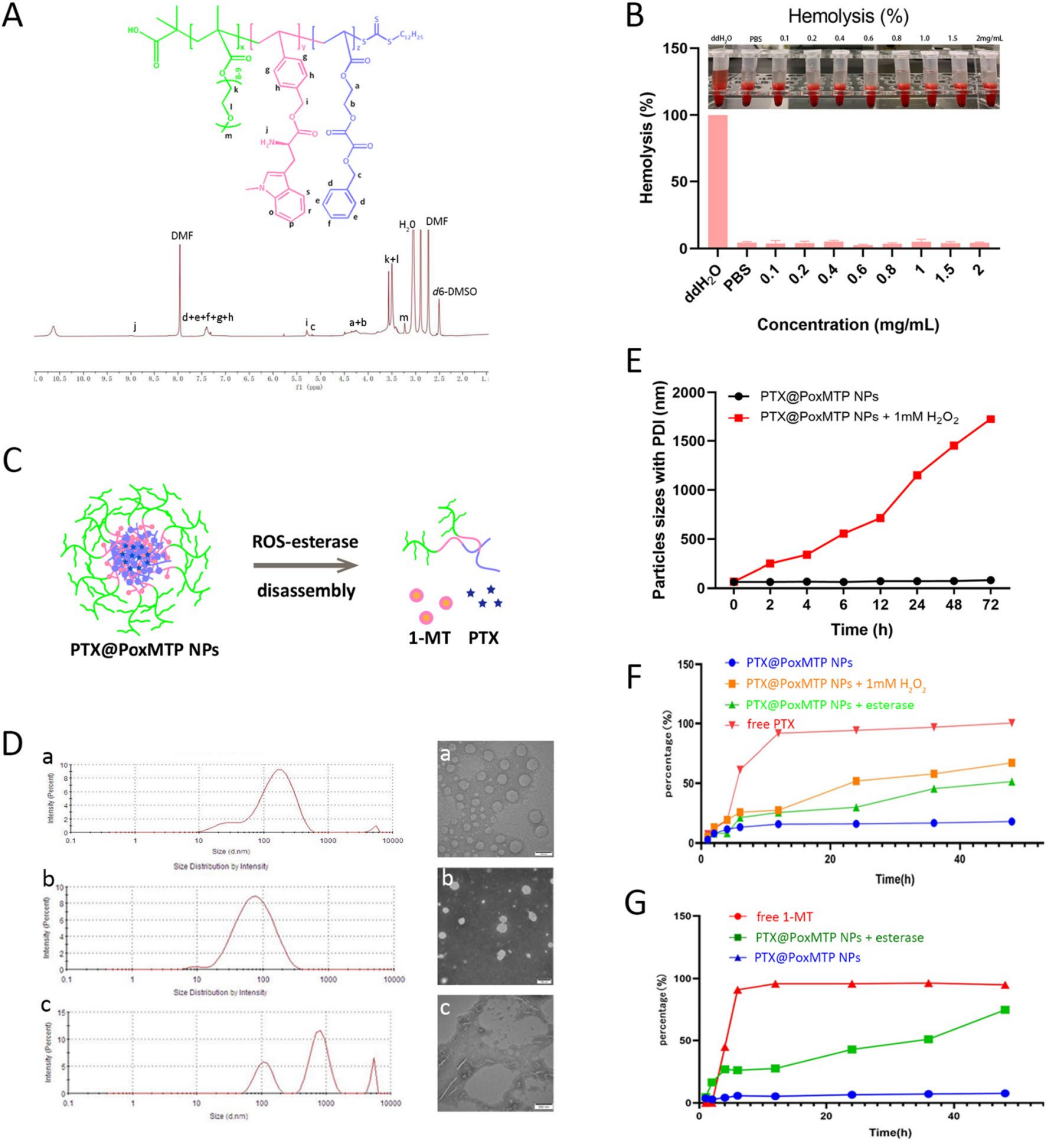
Characterization of the amphilic copolymer and nanoparticles. **A** ^1^H NMR of P[Ph(ox)]-P[MT]-PEG. **B** Hemolytic assay of the copolymer. **C** Schematic illustration of PTX@PoxMTP NPs disassembly in response to ROS and esterase; (**D**) Particle size distribution of blank (a), PTX loaded (b) and H_2_O_2_ treated nanoparticles (c) by DLS and TEM, scale bar = 100 nm; (**E**) Stability of PTX@PoxMTP NPs in the absence and presence of 1 mM H_2_O_2_. (F and G) PTX and 1-MT release under different conditions (n = 3)

The 1-MT prodrug monomer was first synthesized and characterized (Additional file 1: Fig. S1). The ^1^H NMR of 1-MT(Boc) acrylate in CDCl_3_ showed δ = 1.26 (-Boc), 4.13 (-CH-), 5.8 and 5.3 (CH_2_ = CH-) and 7.43 (phenyl), indicating the protection of -NH_2_ and conjugation with the monomer. Subsequentially, phenyl-ox-acrylate monomer with peroxalate ester bond was synthesized and the representative ^1^H NMR result was displayed in Additional file 1: Fig. S2. The peaks at δ = 4.2–4.5 (-CH_2_CH_2_-), 5.2 (-CH_2_-), 5.8–6.4 (CH_2_ = CH-) and 7.4 (phenyl) suggested the successive synthesis of Phenyl-ox acrylate monomer. The polymerization of P[Ph(ox)]-P[MT]-PEG was through reversible addition-fragmentation chain transfer (RAFT) reaction and the ^1^H NMR was shown in Fig. 1A. The peaks at δ = 2.5 and 7.4 represent methylene and phenyl meanwhile δ = 3.5 belongs to PEGMA. The signal peak of 1-MT appears at δ = 5.23 and 7.35, accompanied by the absence of δ = 5.0–5.5, which confirmed the polymerization of these monomers. Furthermore, the disappearance of peaks at δ = 1.25 demonstrated the deprotection of -NH_2_ group (δ = 8.9). The above results suggested that the ROS responsive polymeric prodrug was successfully synthesized. From the integral values of the peak area of the proton in the copolymer, we could calculate the ratio of PEG: P[MT]: P[Ph(ox)] was 2:1.1:1.1**.**

For most antitumor nanomedicines, intravenous injection is the commonest route and thus it must be concerned to estimate their hemolysis activity [36]. Figure 1B displayed that the resultant copolymer achieved negligible hemolytic capability (~ 4%) at experimental concentrations below 2 mg/mL, indicating the acceptable biocompatibility and safety for intravenous administration.

### Characterization of PTX@PoxMTP NPs

As an amphiphilic copolymer, P[Ph(ox)]-P[MT]-PEG can self-assemble into nanoparticles in aqueous solution. PEG form outer hydrophilic shell while lipophilic agents such as PTX can be entrapped into the core through hydrophobic interactions and “π-π” stacking. The physicochemical properties of nanoformulations have shown significant influence on their in vivo biodistribution and therapeutic efficacy [37]. Therefore, particle size, surface charge, morphology, entrapment efficiency and release behavior were carefully investigated. As Fig. 1D shown, blank nanoparticles (PoxMTP NPs) revealed a diameter of 115.3 nm with PDI of 0.452. The introduction of PTX led to a significant decrease in diameter of 62.09 nm with PDI of 0.264, which might be attributed to stronger interactions between PTX and hydrophobic core. TEM images revealed the homogeneous size distribution and spherical appearance of PTX@PoxMTP NPs without visible aggregation, in agreement with DLS results. The zeta potential of PTX@PoxMTP NPs displayed − 0.46 mV, which was comparable to that of blank nanoparticles. Furthermore, the nanoparticles displayed good stability in physiological environment owing to ““π-π” stacking, as evidenced by slight diameter increase but less than ~ 15 nm at storage (Fig. 1E). Next, the encapsulation (EE) and drug loading efficiency (DLE) were measured using HPLC method and the results were shown in Table 1. PTX@PoxMTP NPs achieved EE of 62.5% and DLE of 5.68%, highlighting their potential for hydrophobic cargo entrapment.

**Table 1:** Characterization of PTX@P0.5oxMTP NPs (n = 3)

	Particle size (nm)	PDI	Zeta potential (mV)	EE (%)	DLE (%)
PoxMTP NPs	115.3 ± 3.2	0.452 ± 0.11	− 0.182 ± 0.01	–	–
PTX@PoxMTP NPs	62.09 ± 4.15	0.264 ± 0.08	− 0.461 ± 0.15	62.5 ± 2.14%	5.68 ± 0.37%
PTX@PoxMTP NPs + H_2_O_2_	556.1 ± 40.6	0.791 ± 0.24	–	–	–

In view of the cleavable property of peroxalate ester (ox), we believed that PTX release from the nanoparticles could be observed in an oxidative environment. To investigate the ROS-responsive properties of PTX@PoxMTP NPs, we first monitored their size and morphological changes in the presence of H_2_O_2_ conditions. Figure 1D, E revealed that the incubation with 1 mM H_2_O_2_ led to the disassembly of PTX@PoxMTP NPs with significant increase in particle sizes (~ 550 nm at 6 h) and irregular and swollen appearance against their good stability in physiological conditions. This would facilitate PTX release in response to intracellular ROS accumulation as the disproportion of superoxide dismutase in mitochondria and PTX associated increase activity of NADPH oxidase in tumor cells [38, 39].

Subsequently, the release profiles of these nanoparticles were evaluated under different conditions and the results were illustrated in Fig. 1F, G. Free PTX displayed rapid release into medium with 90% detectable within 10 h. On the contrary, the introduction into nanoparticles significantly reduced PTX release with 11% at 24 h and 12% at 48 h, indicating good stability with no burst release. H_2_O_2_ treatment enabled rapid PTX release from nanoparticles in a controllable way, implying ROS-responsive peroxalate ester cleavage and following nanocarrier’s disassembly. As for 1-MT, the release was much slower with 6% for 48 h whereas liver esterase treatment resulted in a rapid release with prolonged incubation time (79% within 48 h). Meanwhile accelerating PTX release was observed in the presence of esterase, suggesting that 1-MT detachment reduced the stability of the nanoparticles. The above results validated the dual-responsive properties of PTX@PoxMT NPs that enabled controlled cargo release in responses to esterase and ROS conditions while good stability in physiological environment.

### Cellular uptake

**Fig. 2:**
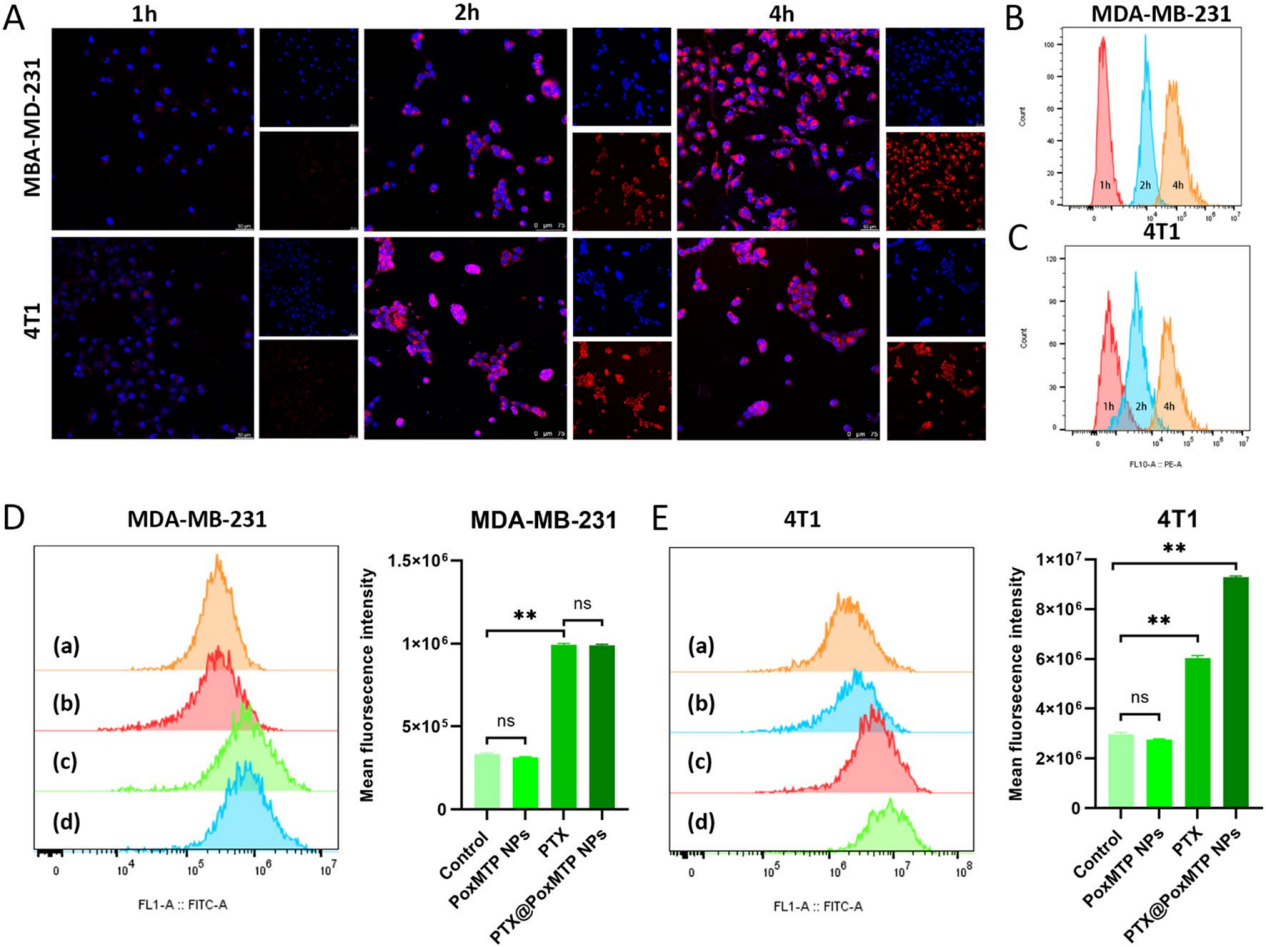
Cellular uptake and ROS assay. **A**–**C** Cellular uptake of nile red (NR) labelled PTX@PoxMTP NPs in MDA-MB-231 and 4T1 cells using CLSM and flow cytometry. (**D** and **E**) Flow cytometry and analysis of ROS production in tumor cells after PTX@PoxMTP NPs treatment. *P < 0.05, **P < 0.001

A major requirement for antitumor effects of most chemotherapeutic agents is dependent on their cellular uptake in tumor cells. In this work, nile red (NR) was used to replace PTX and the cellular uptake was tested using flow cytometry and CLSM observation. As shown in Fig. 2A, a stronger red fluorescence was observed within cytoplasm of MDA-MB-231 and 4T1 cells, suggesting their intracellular internalization while not absorption on the cells’ surface. Particularly, intracellular diffusion of fluorescence signals indicated that ROS induced NR release from the nanoparticles. Flow cytometry analysis present similar results, which revealed a time-dependent internalization of NR@PoxMTP NPs in both tumor cells (Fig. 2B, C). These data implied that PoxMTPs NPs could efficiently deliver hydrophobic agents into the cytoplasm of tumor cells with comparable sizes and zeta potentials. Moreover, intracellular PTX analysis indicated that PTX@PoxMTP NPs significantly improved cellular internalization compared to free PTX (Additional file 1: Fig. S3).

### ROS production

Chemotherapeutics usually generate elevated level of intracellular ROS, which cause oxidative damage and tumor cell death [40]. Therefore, PTX formulations induced ROS production in tumor cells was investigated using DCFH-DA as a fluorescent probe. As shown in Fig. 2D, E, the DCF fluorescence in PoxMTP NPs is comparable to that of control group, indicating that the copolymer or 1-MT had no significant influence on ROS generation. On the contrast, free PTX treatment raise the ROS level with 2 ~ threefold improvements compared to control group in both MDA-MB-231 and 4T1 cells. The introduction of PTX into nanoparticles demonstrated 1.5-fold higher than that in free PTX group in 4T1 cells while MDA-MB-231 cells not. These results indicated that PTX@PoxMTP NPs could promote ROS generation mediated by PTX as a ROS inducer in tumor cells, which might be in part associated with increased activity of cytoplasmic NADPH oxidase [39].

### In vitro cytotoxicity

**Fig. 3:**
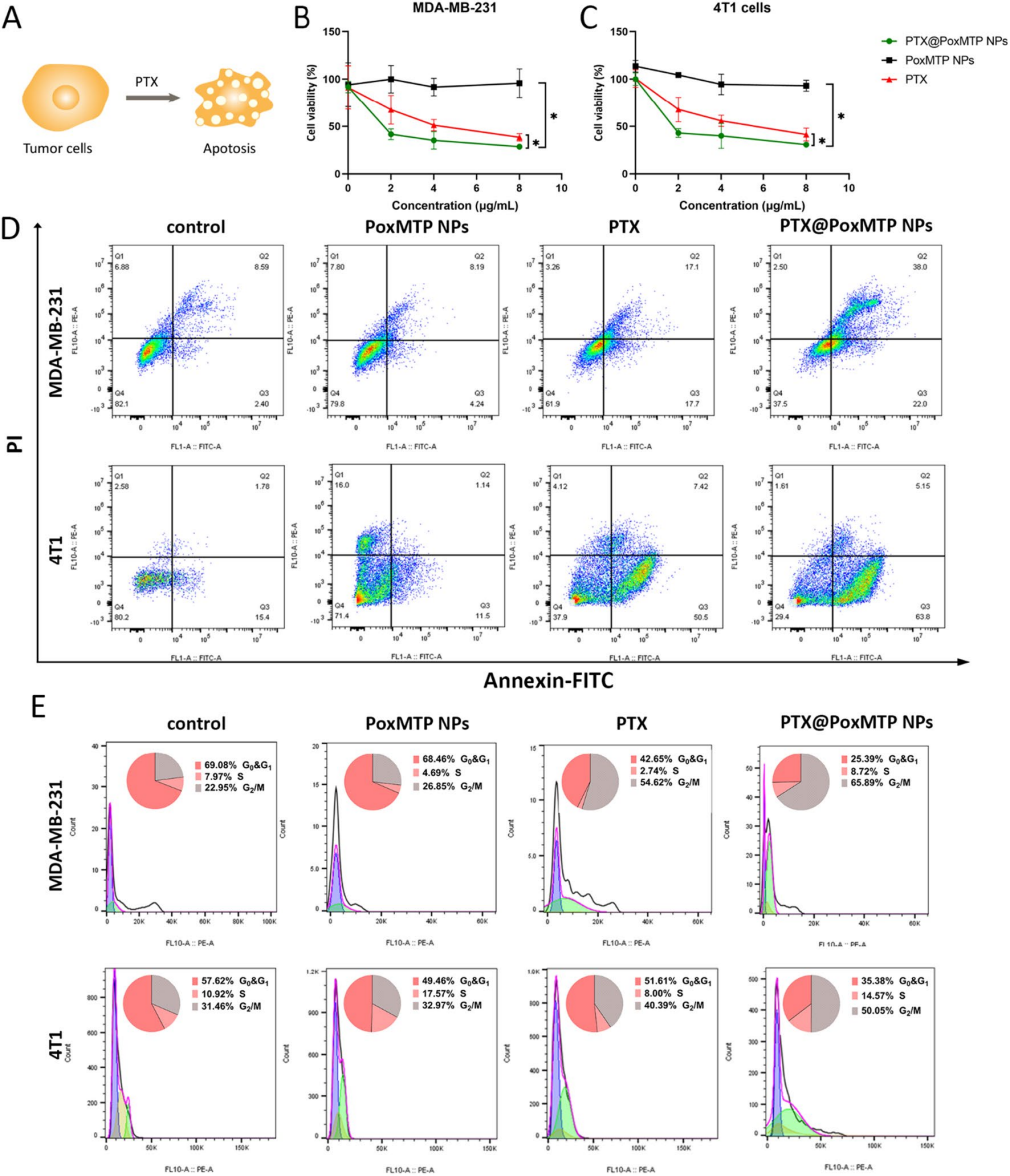
In vitro cytotoxicity assay. **A** Schematic illustration of apoptosis in tumor cells. (**B** and **C**) In vitro cytotoxicity of MDA-MB-231 and 4T1 cells received PTX formulations. (**D** and **E**) Apoptosis and cell cycle analysis in MDA-MB-231 and 4T1 cells treated with different formulations. *P < 0.05

It has been reported that ROS accumulation is an early and crucial step for PTX induced cancer cell death [41]. Next, the cytotoxicity of these formulations was investigated by standard MTT assay. Firstly, the cytotoxicity of 1-MT and PoxMTP NPs were evaluated and the results were shown in Fig. 3B–C and Additional file 1: Fig. S4. No significant difference was observed in the cytotoxicity of 1-MT and PoxMTP NPs (1-MT below 70 μg/mL), both of which demonstrated negligible influence on the proliferation of MDA-MB-231 and 4T1 cells at experimental concentration. On the contrary, PTX predominately suppressed tumor cell proliferation in a dose-dependent manner, which revealed 51.2%and 55.8% survival in MDA-MB-231 and 4T1 cells at concentration of 4 μg/mL. Previous study showed that doxorubicin loaded nanoparticles were less effective than free drug due to incomplete release within tumor cells [42]. Herein, ROS-cleavable peroxalate ester was introduced into the prodrug-based nanomaterials to facilitate intracellular cargo release. As expected, PTX@PoxMTP NPs achieved ~ 1.4-fold higher inhibition effects in MDA-MB-231 and 4T1 cells than free PTX. This may be attributed to efficient cellular uptake and cytosolic release of PTX through a positive feedback loop of “ROS-responsive PTX release and PTX mediated ROS-generation”. Given that NLG919 (another IDO inhibitor) could sensitize B16-F10 cells to PTX in the presence of IFN-γ, it is reasonable to conclude that PTX@PoxMTP NPs can achieve better tumor killing effects in vivo [43]. PTX has been proven to induce intracellular O_2_^−^ and H_2_O_2_ accumulation, ER stress, CRT exposure and tumor cell death whereas the antitumor activity is abolished by N-acetylcysteine (NAC, a ROS scavenger) [39, 44, 45]. However, PTX@PoxMTP NPs displayed significant cytotoxicity to MDA-MB-231 cells thought comparable ROS production in comparison with free PTX, suggesting that PTX can trigger tumor cell death through in part other mechanism.

The IC50 values were also calculated, which was in accordance with MTT results. As illustrated in Additional file 1: Table S1, free PTX demonstrated a IC50 value of 4.159 µg/mL for MDA-MB-231 and 4.118 µg/mL for 4T1 cells. While PTX@PoxMTP NPs markedly reduced the IC50 of 2.134 µg/mL, which was 1.95-fold lower than that of free drug in MDA-MB-231 cells. A same trend was found in 4T1 cells, in which a lower concentration of PTX@PoxMTP NPs (2.053 µg/mL) was required to inhibit their proliferation as compared to free drug. In addition, 1-MT and PoxMTP NPs demonstrated no statistical significance in comparison with control group. These results suggested that PTX entrapment into nanoparticles could increase the cytotoxicity to tumor cells as the well-known killing activity of PTX rather than IDO inhibitor.

### Cell cycle and apoptosis

Apoptosis is a pattern of programmed cell death, that has been proven as the major mechanism for most chemotherapeutic agents [46]. Therefore, Annexin V-FITC/PI staining was used to evaluate apoptotic percentage after different formulation treatments. As shown in Fig. 3D and Additional file 1: Fig. S5, the apoptotic percentage induced by PTX were 17.7% and 50. 5% (early apoptosis) for MDA-MB-231 and 4T1 cells as compared to 2.4% and 15.4% in control group, respectively. This can be explained by that PTX can induce caspase 3 activation and PARP cleavage, etc., which promote breast cancer cells undergoing apoptosis [47]. PTX encapsulation into the nanocarrier achieved higher percentage of apoptotic tumor cells with 1.24–1.26-fold enhancement in MDA-MB-231 and 4T1 cells compared to free PTX, which may be due to efficient cellular uptake and release into cytoplasm. Additionally, the results also established that blank nanoparticles (with only 1-MT) had no significant difference on apoptosis of tumor cells compared to control group, consistent with MTT results.

The cell cycle distribution after different treatments was also estimated through PI staining. Figure 3E showed that the percentage of cells arrested in G_2_/M were 54.62% and 40.39% for MDA-MB-231 and 4T1 cells after PTX treatment, which were higher than that of control group (about 10%). PTX@PoxMTP NPs demonstrated that the numbers of MDA-MB-231 and 4T1 cells arrested in G_2_/M were 65.89% and 50.05%, 1.2-fold higher than that of free PTX, suggesting efficient intracellular PTX release through the “positive feedback loop”. Moreover, PTX formulations achieved obvious reduction in the percentage of tumor cells arrested in G_0_&G_1_ rather than S phase. This was attributed to that PTX could inhibit dynamic reorganization of microtubules so that block cell cycle in G_2_/M and mitosis, hindering replication and division of tumor cells [48]. Meanwhile PoxMTP NPs showed no significant impact on cell cycles as their invisible cytotoxicity in vitro. These results inferred that PTX@PoxMTP NPs could significantly suppress tumor cell proliferation through apoptotic induction and cell cycle arrest.

### Immunogenic cell death

**Fig. 4:**
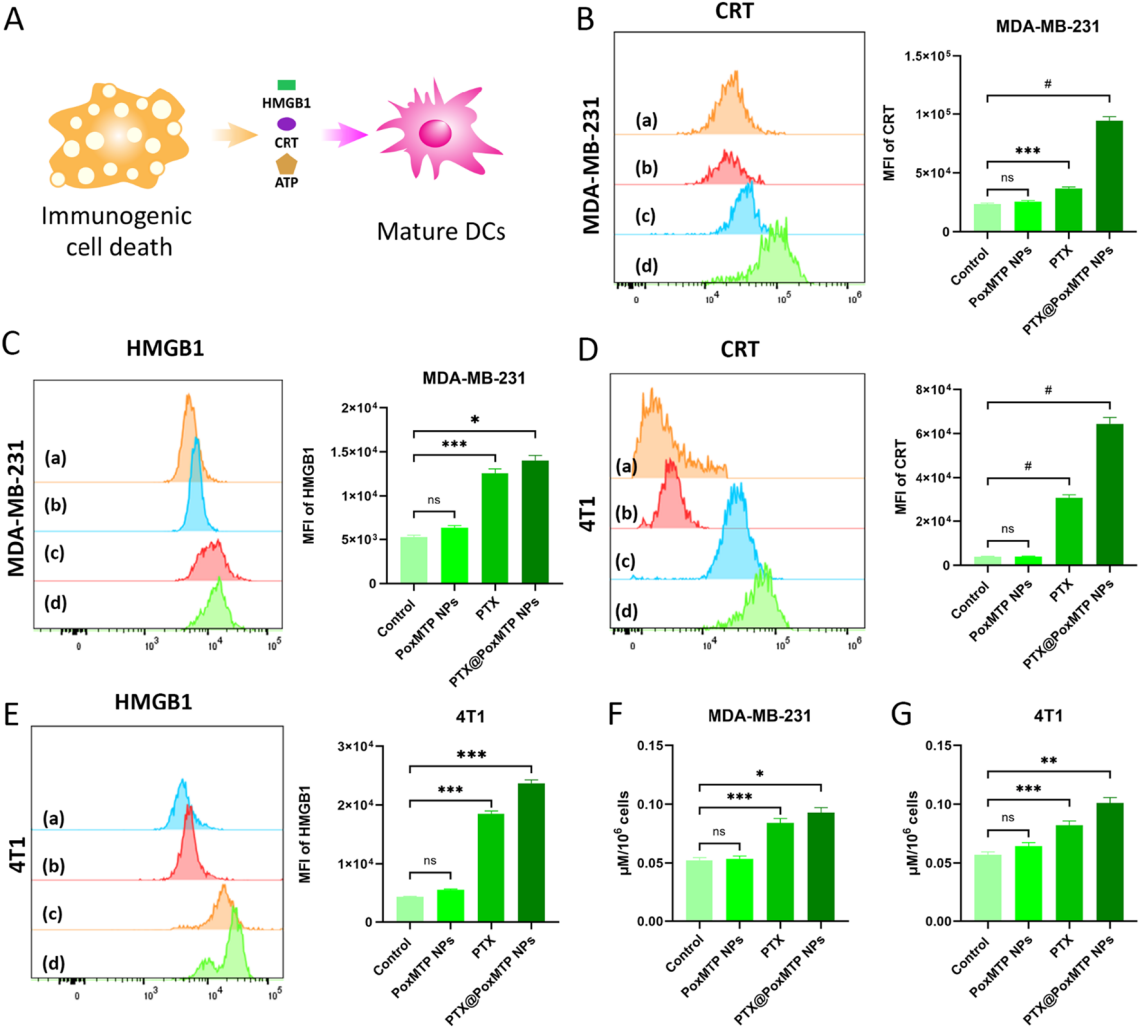
In vitro ICD evaluation. **A** Schematic illustration of tumor cells undergoing ICD. **B**–**E** Flow cytometry and analysis of CRT and HMGB1 expression on tumor cells treated with different formulations (a: control, b: PoxMTP NPs, c: PTX and d: PTX@PoxMTP NPs). **F**–**G** ATP levels in MDA-MB-231 and 4T1 cells in different treatment groups. *P < 0.05, **P < 0.01, ***P < 0.001 and #P < 0.0001

Inspired by recent studies that PTX treatment could induce tumor cell death with intracellular contents release to boost immune responses, specific molecular events such as CRT exposure, HMGB1 and ATP release were evaluated after these formulations’ treatment [49]. As shown Fig. 4B, MDA-MB-231 cells received PTX and PTX@PoxMPT NPs dramatically upregulated the expression of CRT that is consider as an “eat me” signal to activate antigen present cells (APCs) such as dendritic cells. A stronger CRT fluorescence signal was observed in PTX@PoxMTP NPs treated MDA-MB-231 cells, owing to efficient cellular uptake and intracellular self-augmented ROS responsive release of PTX. HMGB1 can promote antigen presentation and the maturation of dendritic cells as a TLR agonist [50]. Meanwhile ATP can act as “find me” signal to promote P2RY2 activation and dendritic cell recruitment into “ICD” sites [51]. Figure 4C, F demonstrated that PTX@PoxMTP NPs augmented HMGB1 and ATP release, 1.12- and 1.1-fold higher than that of free drug in MDA-MB-231 cells. By contrast, no improvements were found in CRT exposure as well as HMGB1 and ATP production after PoxMTP NPs treatment compared to control group. For 4T1 cells, PTX nanoparticles achieved significant enhancements in tumor immunogenicity with upregulated CRT, HMGB1 and ATP, consisting with above results (Fig. 4D, E, G). Overall, the results suggested that PTX@PoxMTP NPs could efficiently induce immunogenic cell death derived from PTX chemotherapy.

### DCs maturation

**Fig. 5:**
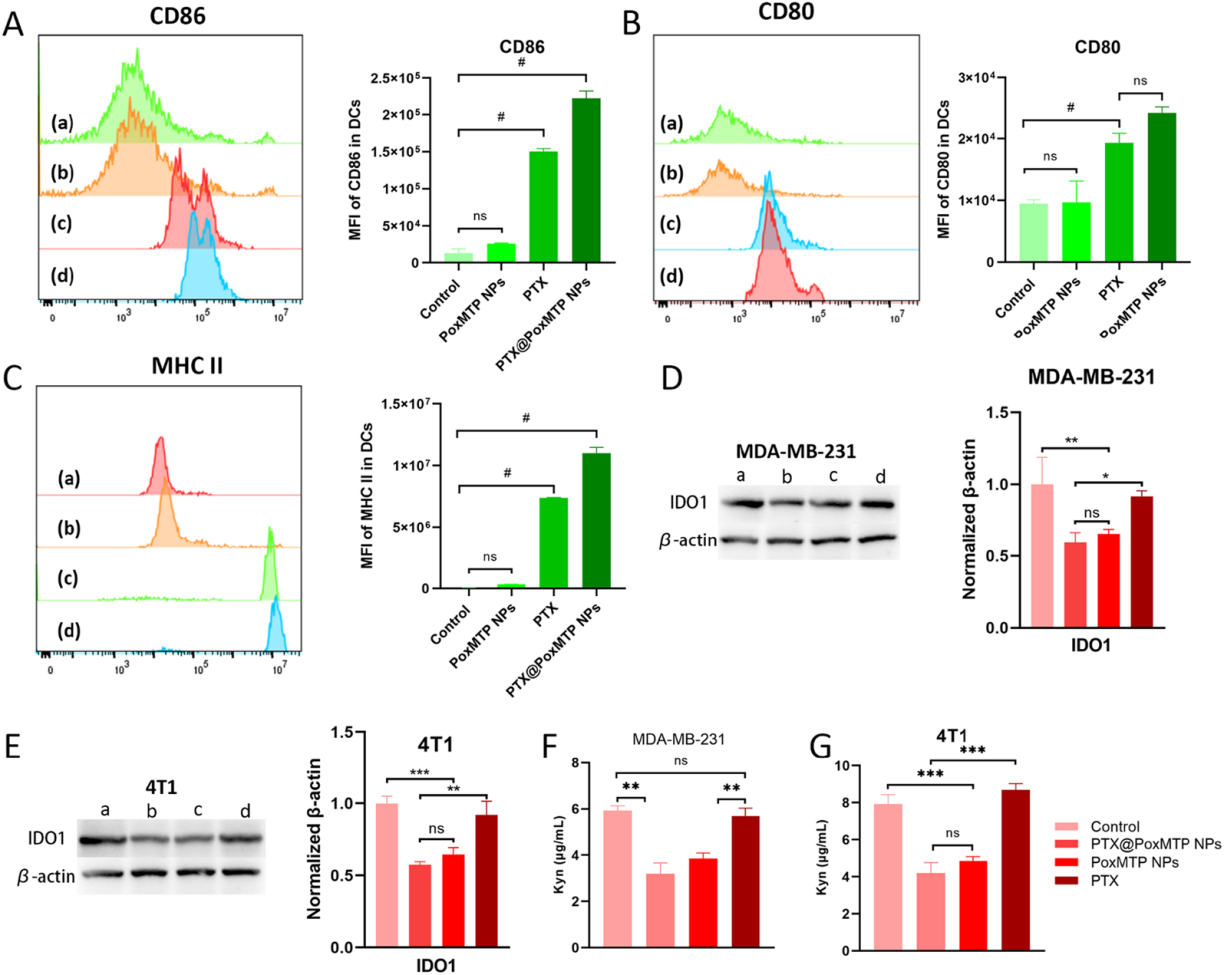
In vitro immune modulation assay. **A**–**C** Mature markers on dendritic cells incubated with 4T1 cells pretreated with different PTX formulations. (a: control, b: PoxMTP NPs, c: PTX and d: PTX@PoxMTP NPs). (**D** and **E**) IDO1 expression analysis after different treatments by WB. (**F** and **G**) Kyn levels in tumor cells received different formulations. *P < 0.05, **P < 0.01, ***P < 0.001 and ^#^P < 0.0001

Dendritic cells play a critical role in the maintenance of innate and adaptive immunity [52]. To further evaluate PTX-induced immunogenicity, BMDCs obtained from Balb/c mice were co-cultured with 4T1 cells pretreated with different formulations and analyzed by flow cytometry. Figure 5A–C illustrated that all PTX formulations evidently promoted mature DCs with varied CD80, CD86 and MHC II upregulation in contrast to control and blank nanoparticles group, verifying efficient immunity in vitro. This may be attributed to PTX mediated ICD that could activate DCs. Moreover, PTX can promote DCs maturation through TLR4 stimulation [53]. Noticeably, PTX@PoxMTP NPs displayed 1.25-, 1.49- and 1.5-fold increases in CD80, CD86 and MHC II expression compared to that of free PTX, further implying the capability of the ROS-responsive prodrug-based carriers in eliciting immune responses. This would augment cytotoxic T lymphocytes mediated killing effects, which have a critical role in tumor suppression.

### IDO inhibition

After maturation, DCs can engulf and present tumor antigens to T lymphocytes and thereby prime immune responses. However, IFN-γ expression from activated T cells can induce IDO1 upregulation, which acutely distracts antitumor immune responses [54]. Thus, it was reasonable to combine chemotherapy mediated ICD with IDO blockade, which has been proven to effectively inhibition IDO expression and augment antitumor outcome [55, 56]. As illustrated in Fig. 5D, E, PoxMTP NPs and PTX@PoxMTP NPs treatment obviously reduced IDO expression compared to control group while PTX not. In addition, the suppression of IDO1 expression was comparable for both PoxMTP NPs and PTX@PoxMTP NPs, might be due to high cellular uptake and esterase triggered drug release. IDO can convert tryptophan into kynurenine, which leads to effector T cells dysfunction and Tregs infiltration. Hence, the level of kynurenine was measured after PTX formulations treatment and the results were shown in Fig. 5F, G. All 1-MT formulations displayed visible reductions in kynurenine production, which was consistent with immunoblot results. Our data validated that PTX@PoxMTP NPs obviously reduced IDO expression and kynurenine production derived from 1-MT. This would contribute to alleviate immunosuppressive tumor microenvironment.

### In vivo distribution

**Fig. 6:**
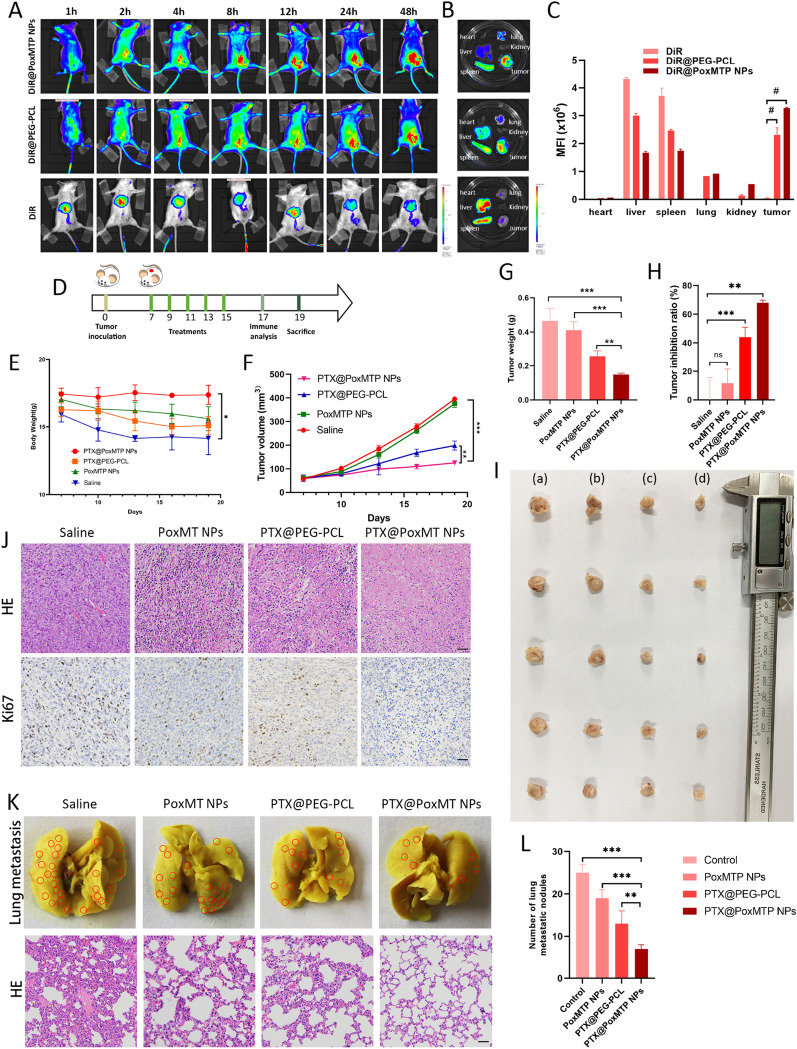
In vivo biodistribution and antitumor evaluation. **A** In vivo biodistribution of DiR labelled nanoparticles in 4T1 tumor bearing mice. **B**–**C** Fluorescence images and analysis of excised major organs and tumors at 48 h after intravenous administration. **D** The timeline of antitumor evaluation. **E** Body weight of mice received different treatments. **F**–**H** Tumor volume, weight and inhibition rate (TGI) in different treatment groups. **I** Representative tumor images from different groups. **J** HE and Ki67 staining of tumor sections. Scale bar = 100 μm. **K**–**L** Representative images, HE staining and metastatic nodules of lungs from different groups. Scale bar = 100 μm. *P < 0.05, **P < 0.01 and ***P < 0.005

In order to investigate the biodistribution of PTX@PoxMTP NPs, 4T1 tumor xenograft mice were intravenously administrated with DiR labeled nanoparticles and monitored by in vivo imaging system. According to Fig. 6A, DiR displayed quick distribution in liver and no detectable fluorescence signal at tumor site for 8 h after intravenous administration. On the contrary, DiR labelled nanoparticles were mainly detected in liver after 1 h administration and thereafter gradually accumulated into tumor sites. At post 4 h administration, major fluorescence signal was observed in tumor rather than other organs. This indicated that nanoscale particles could enable long circulation time (Additional file 1: Fig. S5) and tumor-specific accumulation via enhanced permeation and retention (EPR) effect [57]. Interestingly, DiR@PoxMTP NPs demonstrated higher tumor accumulation rather than DiR@PEG-PCL. Ex vivo images (Fig. 6B, C) further validated the higher tumor distribution of ROS-responsive nanoparticles rather than liver and spleen, which might ascribe for ROS-triggered disassembly and enhanced retention ability [58]. These findings suggested that PTX@PoxMTP NPs would enable tumor-specific accumulation in response to excessive ROS, which would be helpful to decrease unspecific distribution of payloads in normal tissues and minimize side effects.

### Antitumor effects

From above results, it seems plausible that PTX@PoxMTP NPs will deliver cargos to tumor and suppress tumor growth after systemic injection. Herein, the antitumor performance of the ROS-responsive nanoparticles with PTX entrapment was investigated in an orthotopic murine breast cancer model. After 7 days tumor inoculation, Balb/c mice bearing 4T1 tumor received saline, PTX@PEG-PCL, PoxMTP NPs and PTX@PoxMTP NPs treatment at PTX dose of 2 mg/Kg. Figure 6F displayed that monotherapy with PoxMTP NPs had invisible significant reduction in tumor burden as compared to saline group [59]. This might be caused by that poor immunogenicity (also termed as “cold tumor”) led to weak therapeutic response by single immunotherapeutic modulators, though numerous tumor cells died and immune stimulating patterns releases in tumor progression [60]. Growing evidences show that some classical antitumor agents, such as PTX, doxorubicin and oxaliplatin, have triggered immunogenic cell death and generated in situ vaccine-like functions. The combination of chemotherapy and immune modulation would achieve powerful antitumor effects in comparison with monotherapy [61]. PTX@PoxMTP NPs achieved a significant improvement in antitumor effect, much more effective than that of PoxMTP NPs or PTX@PEG-PCL alone, which was likely attributed to synergistic antitumor effects of eliciting immunogenicity and modulating immunosuppressive tumor microenvironment by PTX and 1-MT co-delivery. As illustrated in Fig. 6I, the representative images and weight of tumors from different treatment group were in line with above results. Furthermore, PTX@PoxMTP NPs demonstrated the strongest inhibition ratio of 68.3%, higher than that of PTX with 44.6%, following PoxMTP NPs and saline. It is worth noting that the tumor growth in PTX@PEG-PCL was significantly inhibited as it serves as first line chemotherapeutic drug for breast cancer [62]. To further investigate histological changes after different treatments, tumors were processed for HE and ICH staining and the results were shown in Fig. 6J. It was obviously observed that tumor cells in control group were evenly distributed with intact nucleus and invisible cell damage. For comparison, PoxMTP NPs demonstrated slight chromatin condensation and karyopyknosis in tumor cells, likely due to 1-MT mediated immunosuppressive alleviation. By contrast, PTX formulations displayed strengthened tumor rejection effect with large void space in tumor and cytoplasmic karyorrhexis, validating their exciting therapeutic efficiency. Significantly, PTX@PoxMTP NPs achieved the most apoptotic or necroptotic cells, implying the favorable tumoricidal activity through “self-augmented” PTX release and 1-MT codelivery. Ki67 images revealed that the combination of PTX and 1-MT in one nanocarrier obviously demonstrated the lowest proliferation percentage, once again confirming that PTX@PoxMTP dramatically inhibited tumor progression.

As one of the most aggressive and metastatic carcinomas, lung metastasis of TNBC is the leading cause of mortality [63]. Thus, the anti-metastasis effects of PTX@PoxMTP NPs were investigated through Bouin's and HE staining of lung (Fig. 6K–L). Numerous metastatic nodules were found in saline group, confirming the potent lung metastasis of 4T1 tumor. Conversely, PTX@PoxMTP NPs depicted the least metastatic nodules, following PTX@PEG-PCL, PoxMTP NPs, certainly verifying the satisfactory anti-metastasis activity of PTX and 1-MT combination in one system. In line with above results, lung sections further demonstrated PTX/1-MT nanoparticles with the strongest anti-metastasis capability among these treatment groups. In addition, no significant histological differences were observed in main organs after different treatments compared to saline group (Additional file 1: Fig. S6), in consistent with invisible distinctions of body weight (Fig. 6E). The above results indicated PTX@PoxMTP NPs could significantly inhibit primary tumor and reduce metastatic nodules with minimal systemic toxicity.

### In vivo immune responses

**Fig. 7:**
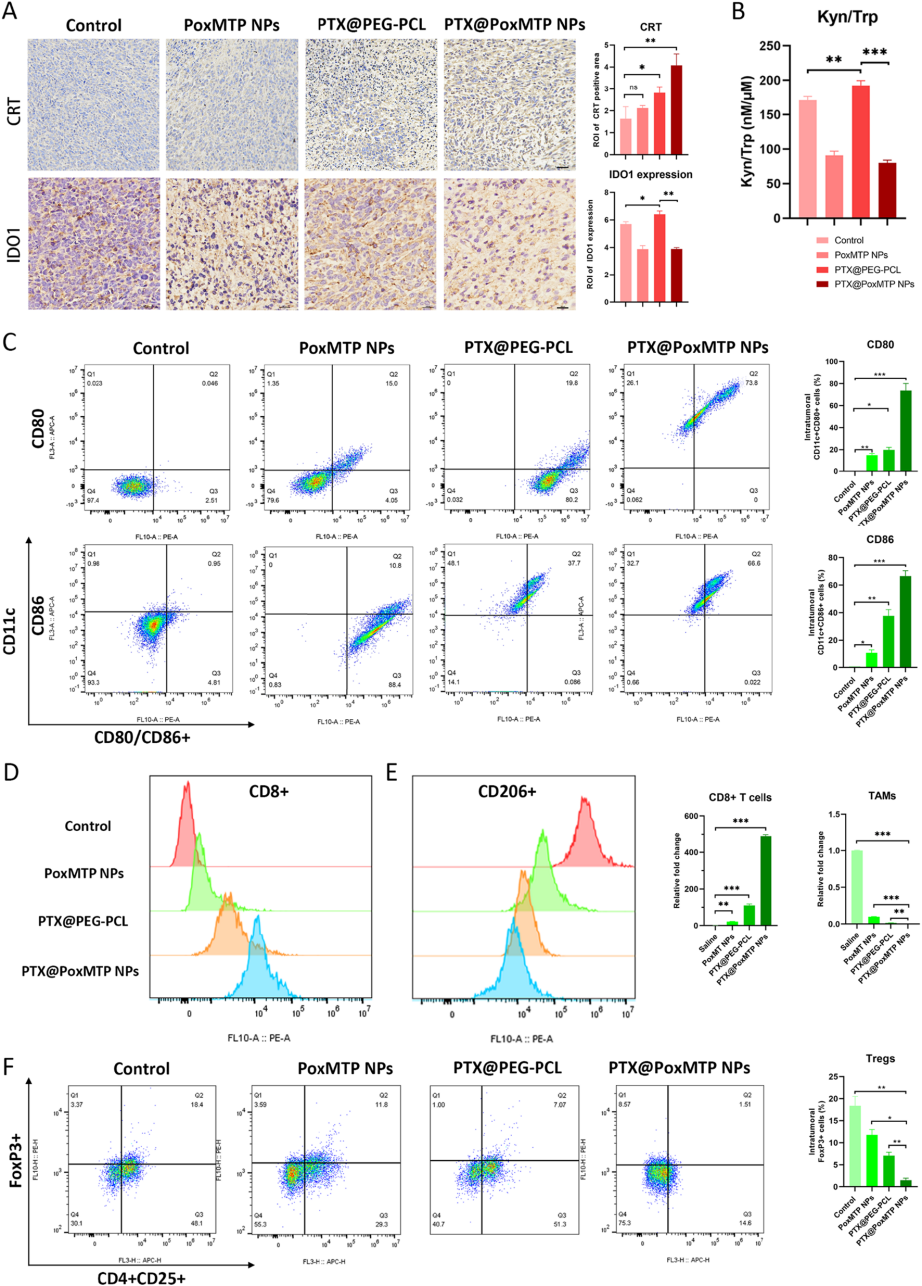
Intratumoral immune components analysis. **A** CRT and IDO1 expression and analysis in tumor sections by ICH staining. Scale bar = 100 μm. **B** Kyn/Trp ratio in tumors by HPLC. **C** In vivo analysis of tumor infiltrating dendritic cells. **D**–**F** Flow cytometry and analysis of CD8 + T cells, TAMs and Tregs infiltration in tumor cells. *P < 0.05, **P < 0.01 and ***P < 0.001

To delineate the role of immunity in PTX@PoxMTP NPs mediated antitumor effects, intratumoral immune responses were analyzed by flow cytometry and ICH staining. Considering that PTX@PoxMTP NPs could induce immunogenic cell death in vitro, in vivo tumor immunogenicity was firstly investigated. As displayed in Fig. 7A, slight CRT expression remained to be observed with control group, suggesting that a substantial number of tumor cells died with immunogenicity. For this reason, intratumoral delivery of immune modulators such as TLR7 agonist resiquimod may achieve local immune activation and tumor regression [64, 65]. However, these DAMPs within tumor microenvironment are prone to be eliminated and tolerated (such as DNase), which, together with immunosuppressive factors, lead to ineffective antitumor immunity. This may be an important cause of distracted antitumor effects of PoxMTP NPs. However, PTX@PEG-PCL treatment led to CRT upregulation, indicating the improvements in tumor immunity after defined chemotherapies. Notably, PTX@PoxMTP NPs effectively facilitated CRT upregulation, higher than that of PTX@PEG-PCL due to self-amplifying ROS triggered efficient PTX release. These data suggested that the introduction of chemotherapeutic agents would be essential to promote tumor immunogenicity.

Next, whether improved tumor immunogenicity mediated by PTX@PoxMTP NPs can activate immune responses was estimated. Figure 7C displayed that PTX@PoxMTP NPs administration achieved improved percentage of CD11c^+^CD80^+^ and CD11c^+^CD86^+^ cells, 3.7- and 1.7-fold higher than that of PTX@PEG-PCL, suggesting successful activation of tumor infiltrating dendritic cells. A possible reason may be PTX mediated indirect and direct DCs stimulation [66]. In addition, we found highest intratumoral CD11c positive cell components after PTX@PoxMTP NPs treatment, which usually demonstrated a strong correlation with tumor-infiltrating T lymphocytes and favorable prognosis in TNBC [67–70]. As shown in Fig. 7D, the combination of PTX with 1-MT further strengthened intratumoral CD8 + T cell infiltration, showing remarkable increase compared to control, PoxMT NPs and PTX@PEG-PCL group, which validated the strong antitumor immunity (Additional file 1: Fig. S7). Interestingly, PoxMTP NPs treatment also achieved significant increased percentage of mature DCs and CD8 + T cells in tumor, which might result from that 1-MT mediated IDO1 inhibition could partly reprogramming DCs and restore tumor immunity [69].

IDO1 expression after different treatment was also evaluated by ICH staining. As shown in Fig. 7A, PTX@PoxMTP NPs and PoxMTP NPs significantly suppressed IDO1 expression, suggesting that the efficient 1-MT delivery can obviously inhibited IDO1 expression within tumor microenvironment. Conversely, PTX administration led to moderate IDO1 upregulation, which may be explained by that PTX-mediated ICD could promote IFN-γ production and following IDO1 overexpression [71]. Fortunately, 1-MT introduction could efficiently restrain PTX-induced IDO1 upregulation. Furthermore, PoxMTP NPs and PTX@PoxMTP NPs revealed significant reduction in Kyn/Trp ratio, which might be associated with anti-metastasis activity of 1-MT (Fig. 6K, L) [72]. This may contribute to reliver immunosuppressive tumor microenvironment.

IDO1 can recruit Tregs and promote their differentiation, which abolishes antitumor immune responses through interfering DCs and CD8 + T lymphocytes. Figure 7F displayed that PoxMTP NPs decreased 61.6% of Tregs infiltration compared to saline group (18.4%), indicating a reduction of Tregs numbers through IDO1 inhibition. Interestingly, the frequency of Tregs infiltration after PTX treatment was reduced to 7.07%, which might be attributed to PTX mediated killing effects in a TLR4 independent fashion [73]. Nevertheless, upregulated IDO expression in PTX-resistant tumor cells may associate with poor responses in advanced breast cancer with single PTX treatment [74]. PTX@PoxMTP NPs achieved the lowest Tregs infiltration within tumor, which might result from PTX-medicated immunogenic killing effects and 1-MT reduced IDO1 expression. The high CD8 + T cells and reduced Tregs can serve as an essential indicator of tumor microenvironment [75]. PTX@PoxMTP NPs demonstrated significant improvement in CD8 + T/Tregs ratios, suggesting a shift from immunosuppressive tumor microenvironment to CD8 + T cells mediated tumor rejection.

IDO can also orchestrate local and systemic immunosuppression by tumor-associated macrophage (TAMs)-Treg axis [76]. TAMs are another important immune cell component with plasticity in tumor microenvironment and most of them exist in immunosuppressive and protumor M2-like subtype. Thus, elimination or reversal of M2-TAMs remains to be a feasible strategy for relieving immunosuppression. From our data, a decreased population of CD206 + macrophage was observed in all treatment groups compared to saline group. Noticeably, PTX@PoxMTP NPs displayed a dramatical reduction of TAMs compared to single treatment with PTX or PoxMTP NPs (Additional file 1: Fig. S8). This might be attributed to synergetic effects of PTX-mediated direct elimination of TAMs and immunosuppressive tumor modulation after IDO inhibition. Moreover, it should be noted that PTX has been shown to directly reprogram M2-TAMs through TLR4 stimulation and delay tumor growth [77]. These results suggested that the self-augmented ROS responsive nanocarrier efficiently modulate tumor microenvironment due to the synergistic therapy of PTX and 1-MT.

## Conclusion

In summary, we developed a self-augmented ROS-responsive nanocarrier for tumor chemoimmunotherapy. The nanoplatform was composed of three polyacrylate blocks including hydrophilic PEG, prodrug (1-MT) and redox-responsive peroxalate ester conjugates meanwhile ICD inducer, PTX was entrapped into the core through hydrophobic interactions and “π-π” stack. After the validation of efficient drug delivery, the nanoplatform achieved positive feedback of “ROS induced PTX release and PTX-mediated ROS production” to promote tumor immunity. Accompanied with enzyme cleavable 1-MT release, PTX@PoxMTP NPs demonstrated significant immunogenic cell death and immunosuppressive tumor microenvironment modulation, which dramatically suppressed primary tumor and reduced lung metastasis. These results suggested the potent potential of co-delivery of ICD inducer and IDO inhibitor by the self-amplifying ROS responsive nanocarrier, which offers a promising strategy for tumor therapy.

## Supplementary Information

**Additional file 1: Figure S1.** 1H NMR of 1-MT(Boc)-acrylate. **Figure S2.** 1H NMR of Phenyl-ox-acrylate. **Figure S3.** Intracellular PTX concentration after 4h incubation (n=3). *P<0.05, **P<0.01. **Figure S4.** In vitro cytotoxicity of 1-MT against MDA-MB-231 and 4T1 cells. **Figure S5.** Apoptosis percentage of tumor cells received different treatments (n=3). **Figure S6.** Plasma PTX concentration at different time after i.v. administration (n=3). **Figure S7.** HE staining of main organs in different treatment groups. Scale bar=100 μm. **Figure S8.** Intratumoral CD8+T cells after different treatments. **Figure S9.** Intratumoral TAMs cells (CD206+) after different treatments. Intratumoral CD8+T cells after different treatments. **Table S1.** IC50 values of PTX formulations against tumor cells (n=6).

## Data Availability

We will support data and materials on request.
