# Supplementary material for: Efficient tumor synergistic chemoimmunotherapy by self-augmented ROS-responsive immunomodulatory polymeric nanodrug
Source: J Nanobiotechnology. 2023 Mar 16;21:93. doi: 10.1186/s12951-023-01842-1 (PMC10018933; doi:10.1186/s12951-023-01842-1)
Supplement: Supplementary file 1 — Additional file 1: Figure S1. 1H NMR of 1-MT(Boc)-acrylate. Figure S2. 1H NMR of Phenyl-ox-acrylate. Figure S3. Intracellular PTX concentration after 4h incubation (n=3). *P<0.05, **P<0.01. Figure S4. In vitro cytotoxicity of 1-MT against MDA-MB-231 and 4T1 cells. Figure S5. Apoptosis percentage of tumor cells received different treatments (n=3). Figure S6. Plasma PTX concentration at different time after i.v. administration (n=3). Figure S7. HE staining of main organs in different treatment groups. Scale bar=100 μm. Figure S8. Intratumoral CD8+T cells after different treatments. Figure S9. Intratumoral TAMs cells (CD206+) after different treatments. Intratumoral CD8+T cells after different treatments. Table S1. IC50 values of PTX formulations against tumor cells (n=6). [file 12951_2023_1842_MOESM1_ESM.docx]

**Efficient tumor synergistic chemoimmunotherapy by self-augmented ROS-responsive immunomodulatory polymeric nanodrug**

Jinxiao Song†, Mingyang Cheng†, Yi Xie, Kangkang Li and Xinlong Zang*

School of Basic Medicine, Qingdao University, Ningxia Road 308, Qingdao, PR China

† These authors contributed equally to this work.

*Corresponding author, e-mail: [zangxinlong@126.com](mailto:zangxinlong@126.com).

**Additional file information**

- 1. **Pharmacokinetics studies**

Pharmacokinetics of various PTX nanoparticles was investigated in Sprague-Dawley rats (SD, 180-200g) obtained from Jinan Pengyue animal Co. Ltd. The rats were randomly divided into two groups (n=3) and intravenously administrated with PTX@PEG-PCL and PTX@PoxMTP NPs at dose of 20mg/Kg. At designed intervals, blood samples were harvested and centrifuged and then the plasma was collected. To analyze PTX content, the plasma was mixed with methanol containing docetaxel as internal standard. The supernatant was collected and dried under nitrogen. The extraction residue was reconstituted in methanol and determined by HPLC-MS.


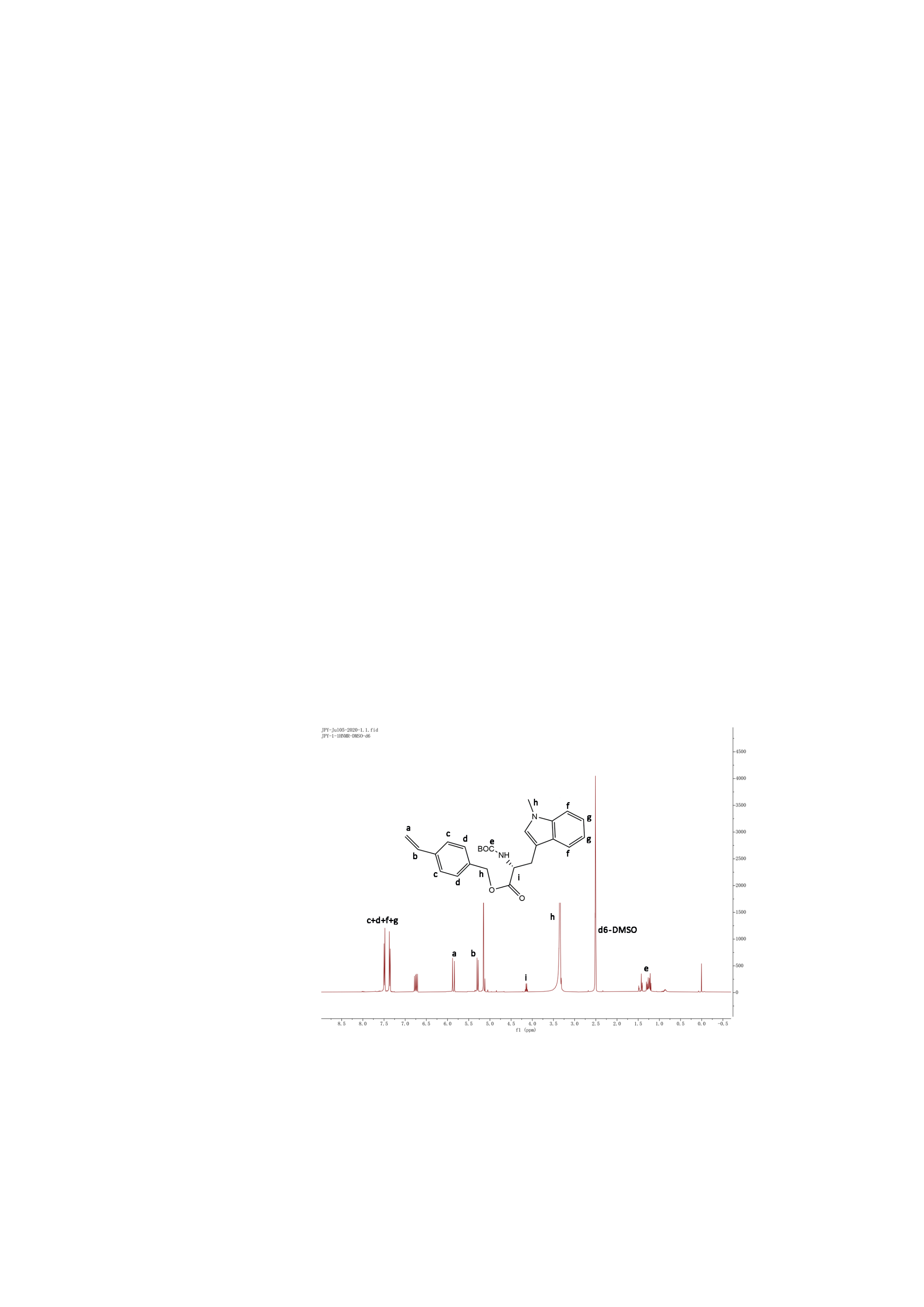


**Figure S1.** ^1^H NMR of 1-MT(Boc)-acrylate.


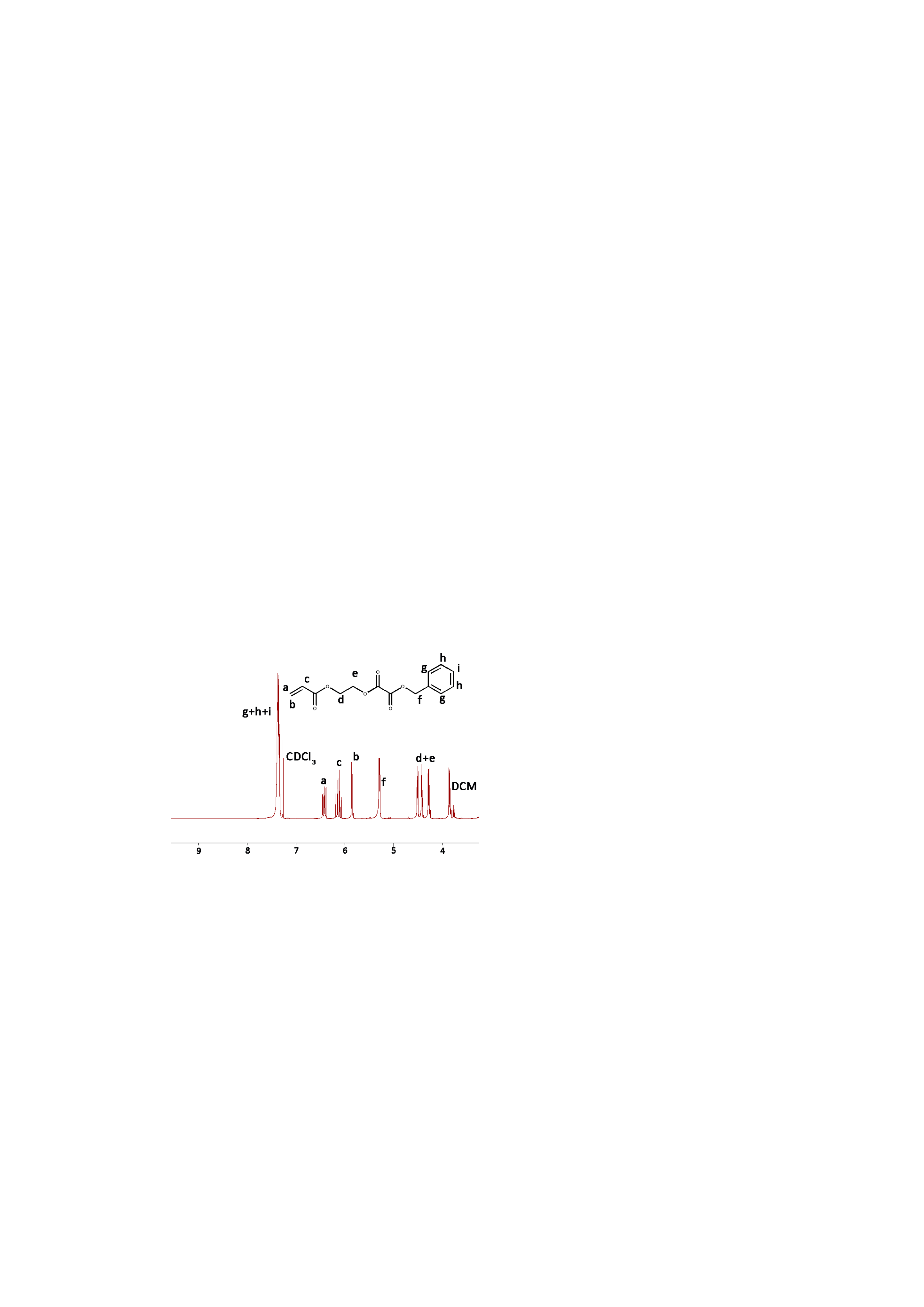


**Figure S2.** ^1^H NMR of Phenyl-ox-acrylate.


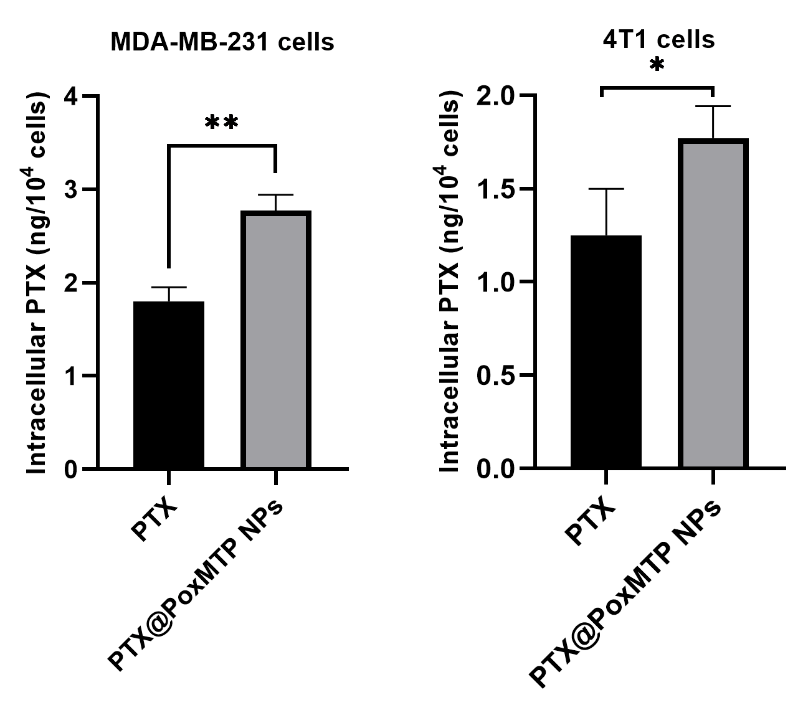


**Figure S3.** Intracellular PTX concentration after 4h incubation (n=3). *P<0.05, **P<0.01.


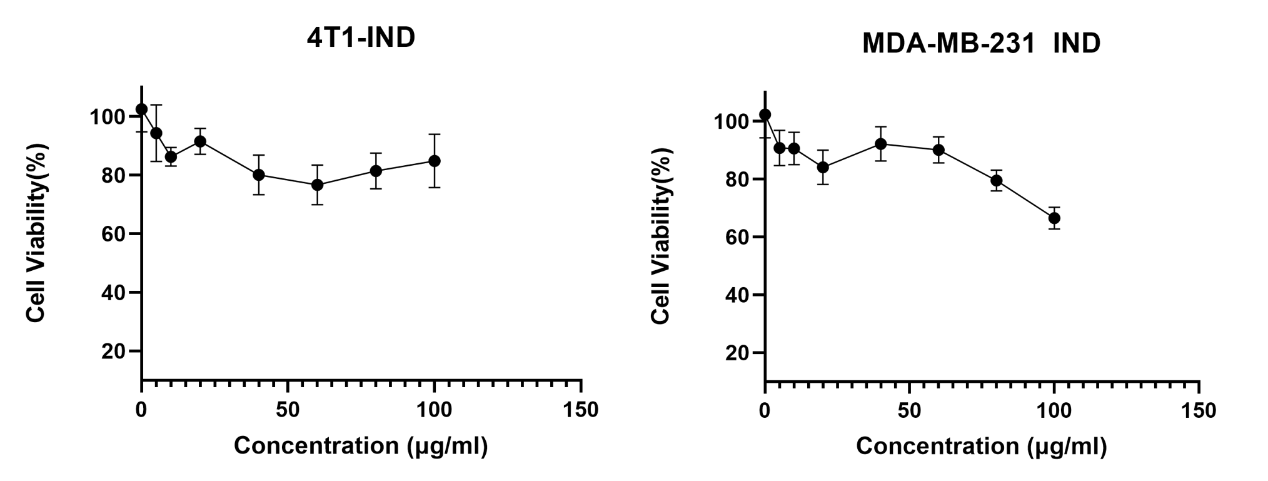


**Figure S4.** In vitro cytotoxicity of 1-MT against MDA-MB-231 and 4T1 cells.


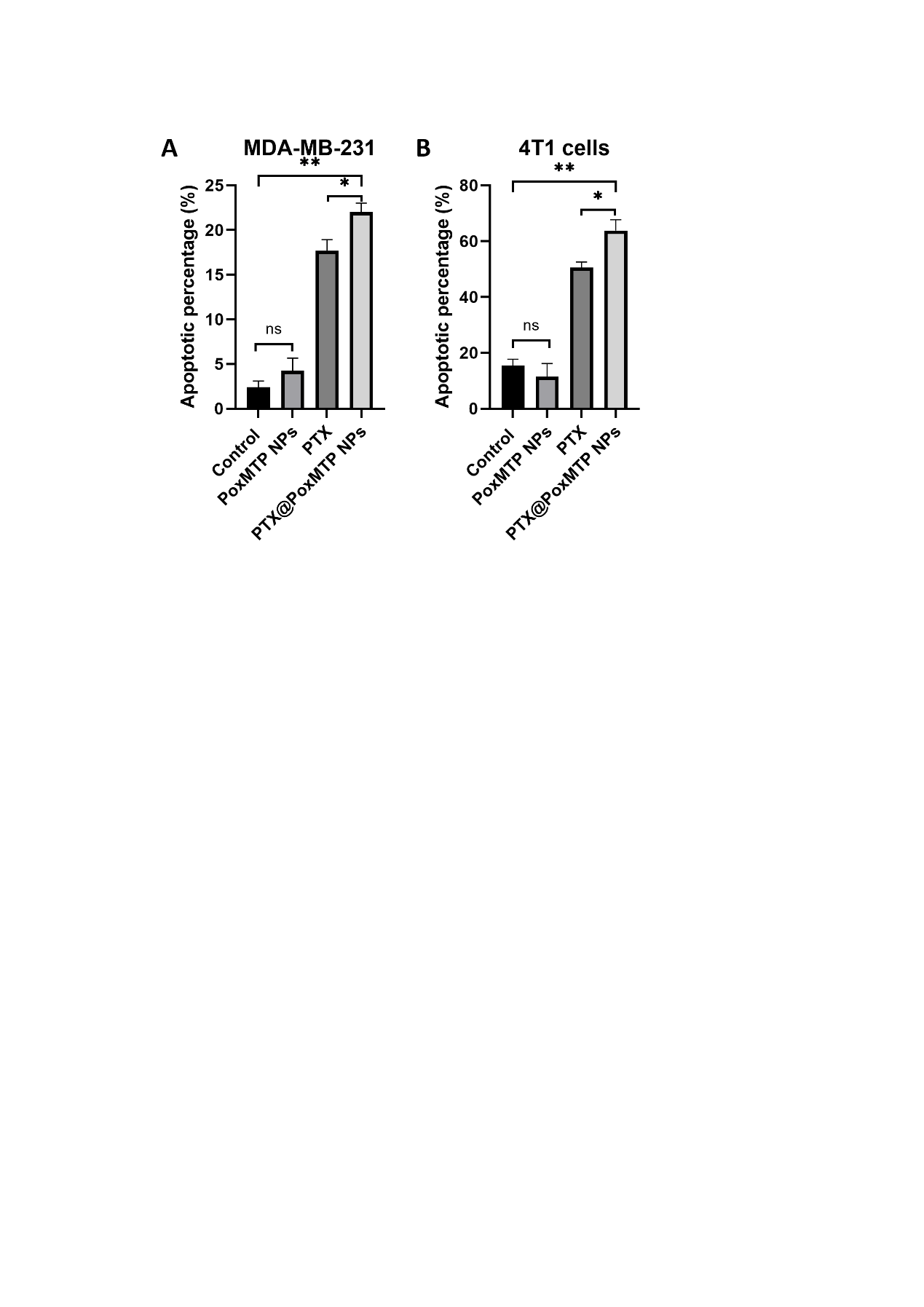


**Figure S5.** Apoptosis percentage of tumor cells received different treatments (n=3).

**Table S1.** IC_50_ values of PTX formulations against tumor cells (n=6).

|  | IC50 (µg/mL) | |
| --- | --- | --- |
|  | 4T1 | MDA-MB-231 |
| 1-MT | -- | -- |
| PoxMTP NPs | -- | -- |
| PTX | 4.118 | 4.159 |
| PTX@PoxMTP NPs | 2.053 | 2.134 |


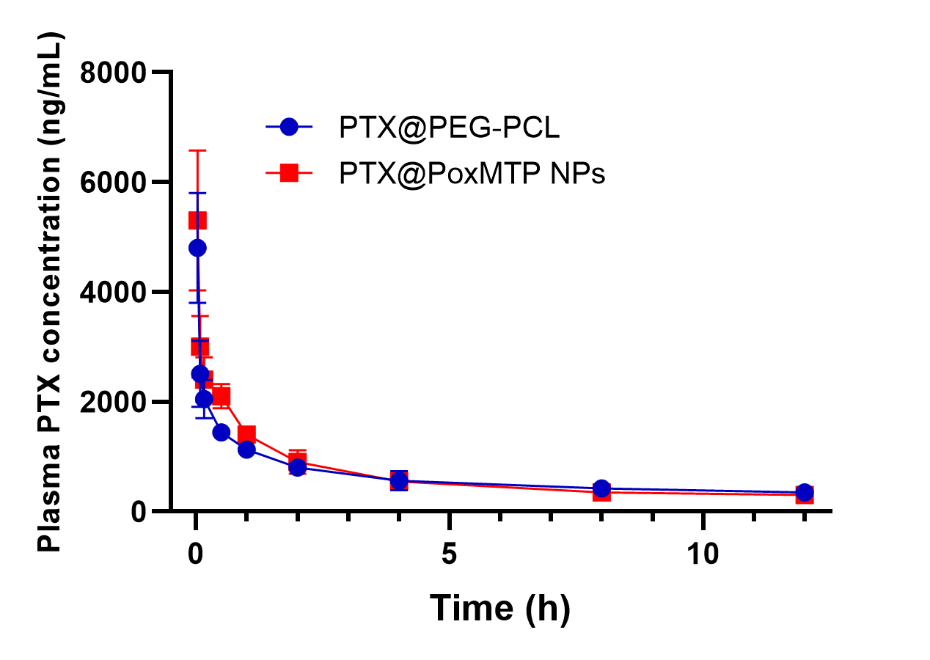


**Figure S6.** Plasma PTX concentration at different time after i.v. administration (n=3).


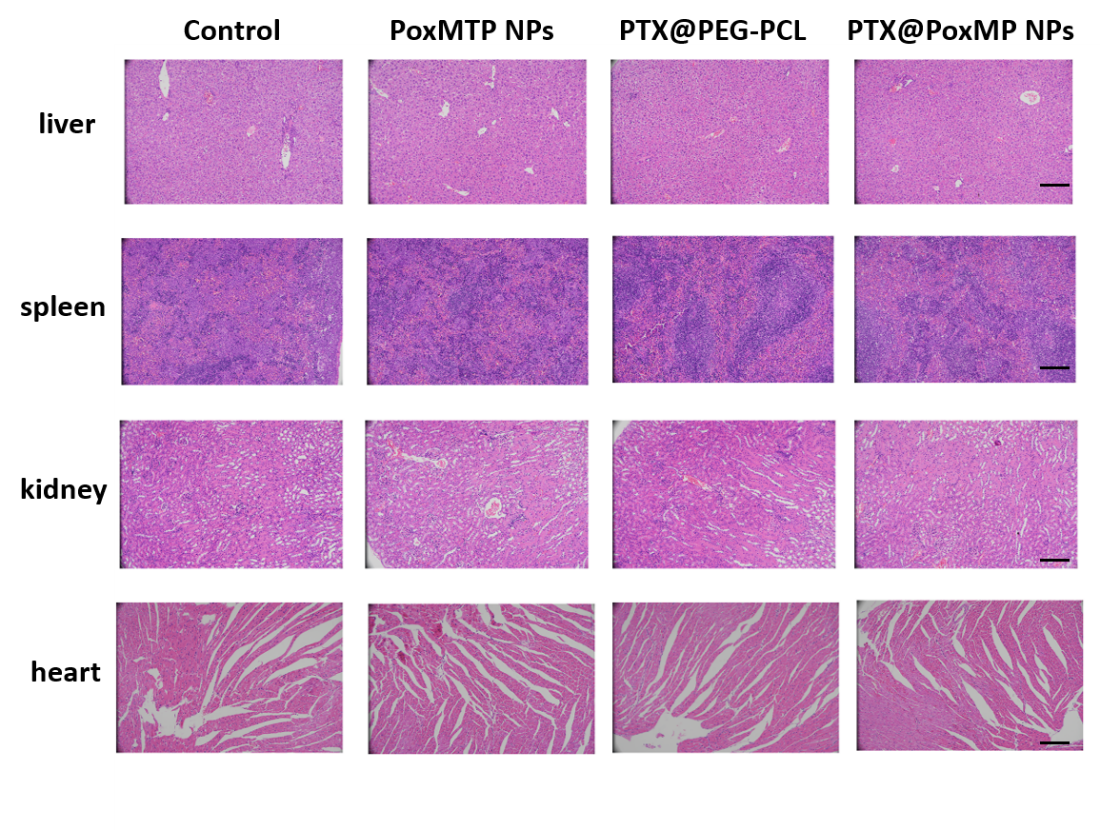


**Figure S7.** HE staining of main organs in different treatment groups. Scale bar=100μm.


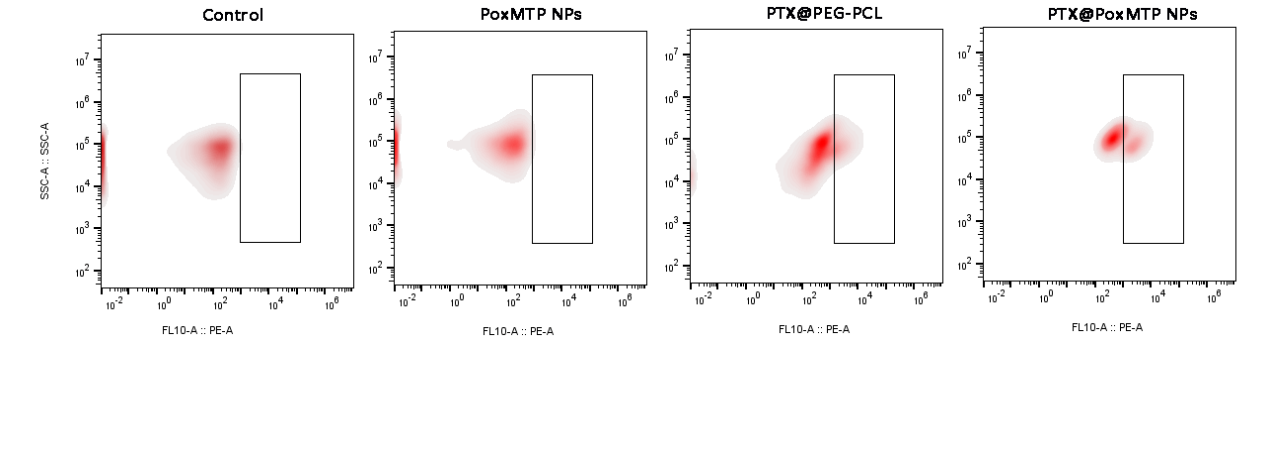


**Figure S8.** Intratumoral CD8+T cells after different treatments.


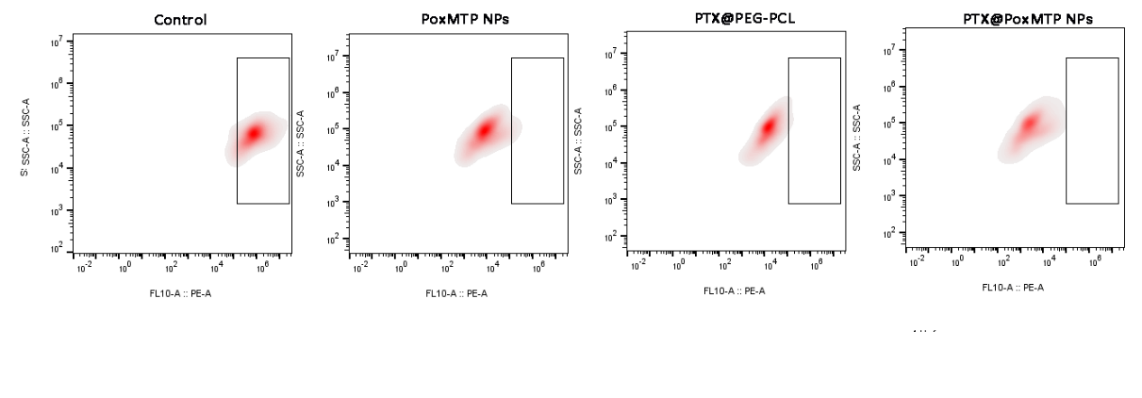


**Figure S9.** Intratumoral TAMs cells (CD206+) after different treatments.
